# Mechanism of salvianolic phenolic acids and hawthorn triterpenic acids combination in intervening atherosclerosis: network pharmacology, molecular docking, and experimental validation

**DOI:** 10.3389/fphar.2025.1501846

**Published:** 2025-01-30

**Authors:** Qu Zhai, Shixi Shang, Zihan Zhang, Lihua Sun, Ying Huang, Shuyi Feng, Qian Wu, Haifeng Cui, Xiaolu Shi

**Affiliations:** ^1^ Institute of Executive Development, China National Medical Products Administration, Beijing, China; ^2^ Beijing Key Laboratory of TCM Basic Research on Prevention and Treatment of Major Disease, Experimental Research Center, China Academy of Chinese Medical Sciences, Beijing, China; ^3^ Beijing University of Chinese Medical, Beijing, China

**Keywords:** salvianolic phenolic acids, hawthorn triterpenic acids, atherosclerosis, network pharmacology, molecular docking, inflammatory regulatory

## Abstract

**Background:**

This study employs network pharmacology and molecular docking methods in conjunction with animal experimentation to elucidate the underlying mechanism by which the combination of salvianolic phenolic acids and hawthorn triterpenic acids (SHC) exerts its therapeutic effect on carotid atherosclerosis (AS) in ApoE^−/−^ mice.

**Methods:**

A network pharmacology research approach was used to predict potential core targets for SHC intervention in atherosclerosis. The predictions were subsequently validated through the implementation of animal *in vivo* experiments. ApoE^−/−^ mice were randomly assigned to three experimental groups, namely, a model group, an atorvastatin group, and an SHC group. After the administration period, the plaque area in the carotid artery and aortic arch, blood lipid levels, malondialdehyde (MDA), superoxide dismutase (SOD), glutathione (GSH), and nitric oxide (NO) content were measured. Additionally, the expression of PI3K, Akt, NF-κB, JNK1, ERK1/2, and p38-MAPK in the aortic arteries was analyzed. Based on the protein expression results, molecular docking was used to predict the binding activity between the core compounds and core targets.

**Results:**

A total of 23 core compounds were identified in SHC, and 55 core targets of SHC were screened as potential targets for intervention in AS. The results of the enrichment analysis indicated that the principal mechanisms through which SHC exerts its effects in AS are associated with lipid metabolism and the PI3K-Akt and MAPK pathways. The results from animal experiments demonstrated that atorvastatin and SHC markedly reduced the area of carotid plaque and downregulated the levels of TC and LDL-C in ApoE^−/−^ mice. The administration of SHC was associated with an increase in SOD activity and a reduction in NO levels in the livers of mice. Furthermore, SHC was observed to downregulate the expression of NF-κB and p38-MAPK in the carotid region. The results of molecular docking demonstrated that the core compounds of SHC, including salvianolic acid A, B, and C, maslinic acid, ursolic acid, and oleic acid, were capable of stably binding to the core targets NF-κB and MAPK14.

**Conclusion:**

It is hypothesized that SHC may reduce lipid deposition and plaque formation in AS by regulating blood lipids, a process that may be closely linked to the inhibition of inflammatory regulator expression, including NF-κB and p38-MAPK.

## 1 Introduction

**FIGURE 1:**
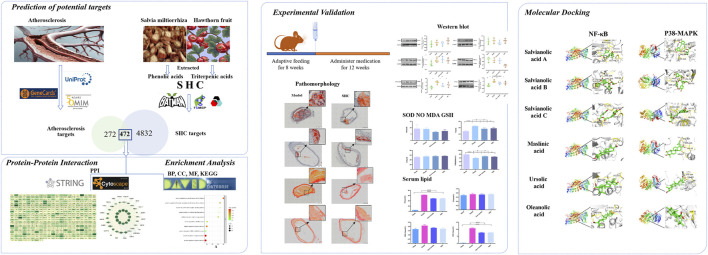
Graphical abstract.

As indicated in the World Health Organization’s 2023 World Health Statistics report, ischemic heart disease and stroke represent the primary and secondary causes of mortality worldwide, respectively. These conditions are identified as the underlying causes of atherosclerotic cardiovascular disease (CVD) (World Heart Federation, 2024). Atherosclerosis (AS) is a chronic inflammatory disease with pathological mechanisms that include abnormalities in lipid metabolism, endothelial dysfunction, activation of immune cells, and cellular stress responses (Gusev and Sarapultsev, 2023). It is notably linked to dyslipidemia. Disturbances in lipid metabolism result in the progressive accumulation of oxidized low-density lipoprotein (oxLDL) within the sub-endothelial matrix, thereby triggering local vascular inflammation and immune responses. This occurs through the binding of oxLDL to scavenger receptors (SRs) expressed on macrophages, endothelial cells, and smooth muscle cells (Borén et al., 2020). Consequently, the objective of clinical interventions aims to modulate lipid metabolism in order to delay the progression of atherosclerotic plaques (Mosalmanzadeh and Pence, 2024). Although research indicates that statin-mediated reduction of LDL can effectively control plaque progression, discontinuation or intermittent administration of statins is detrimental to plaque control (Wu, X et al., 2021). Adverse reactions, characterized by elevated transaminases indicating liver damage and elevated creatine kinase indicating rhabdomyolysis, have been observed in patients receiving statin therapy at doses reaching 40–80 mg (Shavadia et al., 2021). As patients with AS typically require continuous medication, ensuring the safety of the drugs they are taking is of utmost importance. Therefore, the objective of this study is to identify a safe alternative therapeutic option that can effectively improve lipid profiles while potentially exerting potential anti-atherosclerotic effects. Prior research has indicated that the Chinese herbs *Salvia miltiorrhiza* and hawthorn fruit may offer therapeutic benefits in the management of cardiovascular events. The combination of *Salvia miltiorrhiza* and hawthorn fruit has demonstrated efficacy in the treatment of inflammatory conditions, oxidative stress, lipid regulation, and vascular protection. The potential for these herbs to serve as a preventive and therapeutic measure for AS has been demonstrated by several studies (Li ZM et al., 2018; Li H et al., 2022; Wu Y et al., 2020; Li D et al., 2021; Sureda et al., 2021). Phenolic acids, the primary water-soluble constituents of *Salvia miltiorrhiza*, have been demonstrated to possess antioxidant and cardiovascular protective effects (Shen et al., 2018). Triterpene acids, a significant fat-soluble component of hawthorn fruit, have been demonstrated to possess anti-hyperlipidemic and anti-inflammatory properties, as well as the capacity to enhance coronary blood flow (Cui et al., 2024). The anti-atherosclerotic effect of the water-soluble crude extract of the combination of *Salvia miltiorrhiza* and hawthorn fruit has been previously reported (Zhang et al., 2019). However, it is challenging to enrich the lipid-soluble constituents, such as triterpene acids, in hawthorn through aqueous extraction. Network pharmacology is an essential component of systems biology. Its comprehensive and holistic approach to drug–drug interactions is consistent with the fundamental principles of traditional Chinese medicine (Zhao Y et al., 2023). This field of study illuminates the function of traditional Chinese medicines in regulating the organism’s systemic network. To further investigate whether the combination of active ingredients of *Salvia miltiorrhiza* and hawthorn fruit could have an anti-atherosclerotic effect and to clarify their mechanism, the present study was conducted using SHC through a combination of network pharmacology and animal experiments. Figure 1 illustrates the research flowchart.

## 2 Material and methods

### 2.1 Network pharmacology

Screening and target prediction of SHC core compounds were performed by integrating the PubMed database (https://pubmed.ncbi.) with the Traditional Chinese Medicine Systems Pharmacology Database and Analysis Platform (TCMSP, http://tcmspw.com/tcmsp.php); the core compounds of SHC were identified using this approach. The TCMSP database, BATMAN database (http://bionet.ncpsb.org.cn/batman-tcm/), and SwissTargetPrediction database (http://www.swisstargetprediction.ch/) were employed to identify the gene targets of the compounds. Subsequently, the aforementioned targets were validated using the UniProt database (https://www.uniprot.org/).

The collection of SHC-AS intersection targets was obtained by querying the GeneCards (https://www.genecards.org/) and OMIM (https://www.omim.org/) databases with the keyword “atherosclerosis,” and the results were refined using the UniProt database (https://www.uniprot.org/) to identify disease targets. To ensure the accuracy of the disease target identification, the “retrieve/ID mapping” function was employed. The intersection of SHC-AS targets was identified using Venny 2.1 (https://bioinfogp.cnb.csic.es/tools/venny/).

Network construction and core target analysis: The core targets were subjected to screening, and protein–protein interaction (PPI) network diagrams were constructed using the STRING database (https://cn.string-db.org/) and Cytoscape 3.7.1 software.

Enrichment analysis: Gene Ontology (GO) analysis and Kyoto Encyclopedia of Genes and Genomes (KEGG) pathway analysis were conducted on the core targets using the DAVID database (https://david.ncifcrf.gov/). The results were presented in the form of bubble diagrams, providing a visual representation of the data. The threshold for statistical significance was set at *P* < 0.05, and the species selected was *Homo sapiens*. The GO analysis included three domains, namely, biological process (BP), cellular component (CC), and molecular function (MF).

### 2.2 Extraction of SHC components

Extraction of total phenolic acids from *Salvia miltiorrhiza*: The dried root and rhizome of Salvia miltiorrhiza Bge. were obtained. Subsequently, a 4% gelatin solution was added and allowed to stand for 12 h, after which it was filtered. Subsequently, the solution was diluted with ethanol to a concentration of 70% alcohol and allowed to stand for 12 h prior to filtration. An equal volume of 60% ethanol was then added to the precipitate, which was allowed to stand for 12 h and then filtered. The combined filtrate was concentrated to a relative density of 1.23 (50°C) with dilute hydrochloric acid at pH 2–3. Thereafter, an equal volume of water-saturated ethyl acetate was employed for three rounds of shake extraction. Subsequently, the remaining extract was adjusted to a pH value of 5 with sodium hydroxide in order to facilitate the recovery of the ethyl acetate. Subsequently, the resulting extract was subjected to spray drying (Jiang et al., 2022).

Extraction of total triterpenic acid from hawthorn: Hawthorn (Rosaceae, *Crataegus pinnatifida* Bge.) was harvested, ground into a coarse powder, and extracted with 80% ethanol by reflux for two cycles. In the initial stage, the specified amount was increased by a factor of 8, while in the subsequent stage, the specified amount was increased by a factor of 4. Each stage was conducted for a duration of 30 min. The combined filtrate was filtered, and the ethanol was recovered under reduced pressure and concentrated. Subsequently, water was added and stirred to wash the clear paste three times until the pH of the wash solution reached 6. Static filtration was then performed, water was discarded, and the precipitate was spray-dried to yield a dry powder. The resulting dry powder was mixed with four times the amount of ethyl acetate and immersed at 60°C for 30 min. Subsequently, the mixture was filtered, and the retained residue was refluxed three times with ethyl acetate, with each refluxing step involving the addition of 10 times the amount of ethyl acetate. Each of the first, second, and third refluxes was conducted for a period of 0.5 h. Subsequently, the filtrate was filtered, and the resulting solution was collected for further processing with ethyl acetate and vacuum drying. Subsequently, the product was then obtained through vacuum drying (Zhang L et al., 2020).

SHC mixture preparation: A solution was prepared by mixing the total phenolic acid extract of *Salvia miltiorrhiza*, hawthorn total triterpenic acid extract, and microcrystalline cellulose in a ratio of 2:1:2. This solution was then sprayed into ethanol with stirring, forming particles, and subjected to drying and storage.

The comprehensive extraction methodologies for salvianolic phenolic acids, hawthorn triterpenic acids, and SHC are outlined in the supplementary document (Supplementary Material 1, Extraction of SHC Components).

The chemical composition of SHC for each component was confirmed through UHPLC-MS analysis (Supplementary Material 2, Chemical Composition Analysis of SHC).

### 2.3 Animal experiments

#### 2.3.1 Experimental animals

SPF-grade male ApoE^−/−^ mice and C57BL/6Cnc mice, aged 7 weeks, were obtained from Beijing Vital River Laboratory Animal Technology Co. Ltd., a facility licensed by the Beijing Administration for Industry and Commerce (license number SCXK (Beijing) 2016-0006) and has been granted animal qualification certificates (numbers 11400700379466 and 11400700379467). The animals were housed in the Medical Experimental Center of the Chinese Academy of Sciences under the following conditions: temperature 20–22°C, relative humidity 65%–70%, light cycle 12 h/12 h, and *ad libitum* access to food.

The normal diet was purchased from Beijing Vital River Laboratory Animal Technology Co. The high-fat diet (Batch No. MD12015A) was purchased from Jiangsu Medison Biomedical Co. High-fat diet formula: casein (19. 47%), corn starch (4.99%), maltodextrin 9.98%, sucrose (33.76%), cellulose (4.99%), corn oil (0.99%), anhydrous milk fat (19.97%), cholesterol (0.50%), and total minor ingredients (5.35%).

The C57BL/6Cnc mice were acclimatized for a period of 1 week, and the C57BL/6Cnc mice constituted the sham group and were fed a normal chow diet. The ApoE^−/−^ mice were randomly divided into three groups, namely, a model group, an atorvastatin group, and an SHC group. The mice were fed a continuous high-fat chow diet after being sorted by body mass.

#### 2.3.2 Drug administration

The mice in each group were started on the drug. Atorvastatin was supplied in the form of 10 mg tablets, with the batch number T80151. In addition, the active ingredient SHC was extracted and mixed by Guiyang Xintian Pharmaceutical Co, Guiyang, China.

The drug was prepared as a suspension and administered via gavage. Atorvastatin was administered at a concentration of 1.3 mg kg^−1^, and SHC was administered at a concentration of 300 mg kg^−1^. The volume of the administered drug was 0.1 mL/10 g. The corresponding volume of the microcrystalline cellulose solution was administered to the sham and model groups (200 mg kg^−1^) over a 12-week period.

#### 2.3.3 Pathomorphology

The excess adipose tissue of the carotid arteries of mice in each group was excluded, fixed with OCT fixative gel, and then placed in a refrigerator at −80°C for freezing. Thereafter, the tissue was sliced. The slices were fixed in the vertical direction, with the distal end of the heart serving as the point of origin and the point where the double-branched blood vessels merged into a single-branched blood vessel as the point of termination. The thickness of the slices was 8 μm, and the slices were subjected to VG and oil red staining and histopathological observation. The analysis of the plaque area was conducted using ImageJ software.

#### 2.3.4 Western blot

The total protein was extracted from the thoracic aorta and abdominal aortas of each group of mice; the protein concentration was determined by the BCA (bicinchoninic acid) method, and each protein sample was diluted to a uniform concentration. The following proteins were used to detect protein expression: JNK1, PI3K, ERK1/2, Akt phospho-t308, NF-kβ, and p38-MAPK (detailed information on the reagents can be found in Supplementary Material 3, Reagents).

#### 2.3.5 Serum lipid, inflammation, and oxidative stress markers assay

The objective is to ascertain the serum triglyceride (TG), total cholesterol (TC), low-density lipoprotein cholesterol (LDL-C), and high-density lipoprotein cholesterol (HDL-C) levels of mice in each group. The levels of malondialdehyde (MDA), nitric oxide (NO), superoxide dismutase (SOD), and glutathione (GSH) in the livers of mice in each group were determined. Standard curves for the working solutions were constructed separately, and absorbance was measured using an enzyme marker after homogenizing the liver tissue and calculating the concentration. Total GSH assay kit, total SOD activity assay kit, lipid oxidation (MDA) assay kit, and total nitric oxide assay kit were used for detection (detailed information on the reagents can be found in Supplementary Material 3, Reagents).

### 2.4 Molecular docking

By integrating the core targets identified by network pharmacology with the Western blot results, the target with the highest degree of relevance was selected, and the target protein underwent pre-processing using PyMOL software. Molecular docking was performed between the target protein and SHC active compounds based on AutoDock Tools 1.5.6 software, and the minimum binding energy required for docking between the target protein and SHC active compounds was obtained and visualized using PyMOL software. PyMOL software was used for visualization. Intermolecular interactions with binding energies ≤−5.0 kJ/mol were considered to represent relatively strong binding.

### 2.5 Statistical methods

Data were analyzed and processed using SPSS 20.0 statistical software. Experimental data were expressed as ( $\bar{x}\;\pm s$ ), and data between multiple groups were analyzed using the one-way analysis of variance (ANOVA) test. The difference was considered statistically significant at *P* < 0.05.

## 3 Results

### 3.1 Network pharmacology results of SHC

#### 3.1.1 SHC core compounds

Previous literature has identified *Salvia miltiorrhiza* as containing 190 polyphenolic acids and 7 potential new polyphenolic acids (Wu Y. et al., 2020) and hawthorn fruit as containing 6 triterpene acids (Li D. et al., 2021; Sureda et al., 2021). These 203 compounds were entered into the TCMSP database, and 23 SHC core compounds (19 salvianolic phenolic acids and 4 hawthorn triterpenoids) were obtained on the basis of drug-likeness (DL) ≥ 0.18 and oral bioavailability (OB) ≥ 30%, as shown in Supplementary Table 1.

#### 3.1.2 SHC gene targets

**FIGURE 2:**
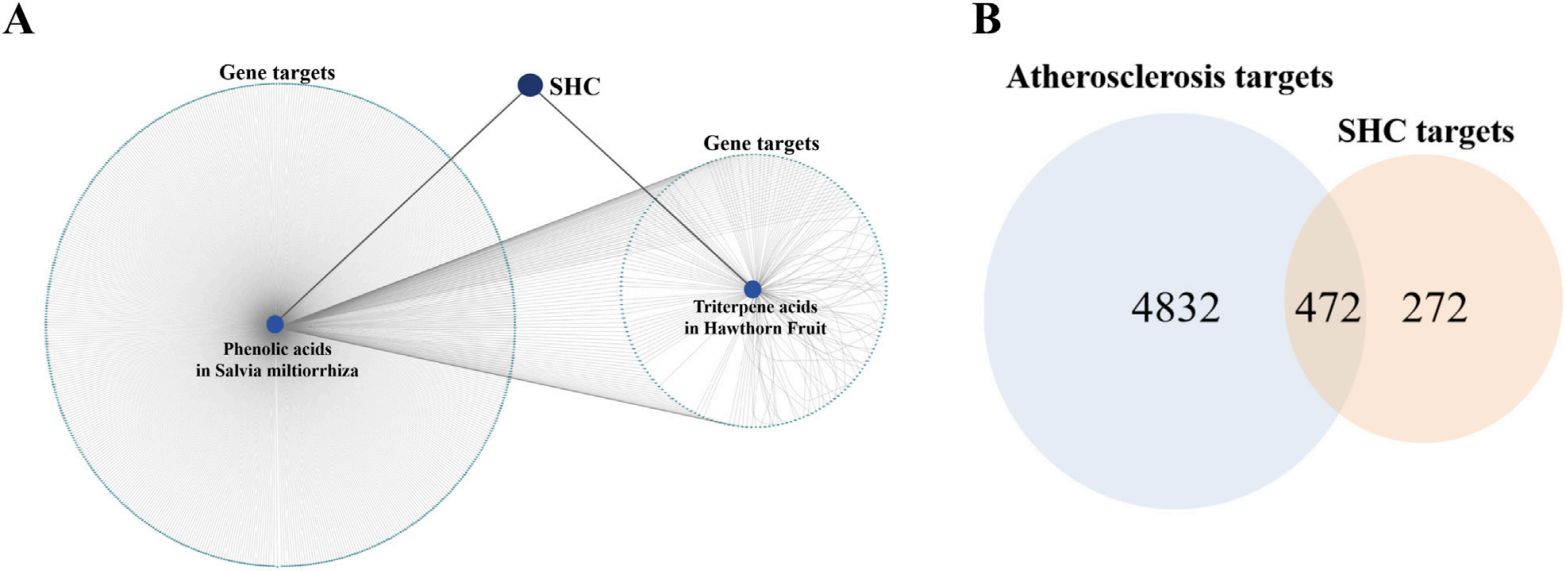
“SHC–Salvia miltiorrhiza phenolic acids & hawthorn triterpenic acids–Gene targets” network diagram and Venn diagram. [Note: **(A)** There are 680 gene targets of *Salvia miltiorrhiza* phenolic acids and 151 gene targets of hawthorn triterpenic acids; after removing the duplicate gene targets, 747 gene targets of SHC were obtained. **(B)** There are 5,304 gene targets of AS, 747 gene targets of SHC, after removing the duplicate gene targets, 472 gene targets were obtained.]

The names, Canonical SMILES, and InChI of the abovementioned 23 compounds were entered into the TCMSP database, BATMAN database (score cutoff ≥ 20 and P-value < 0.05), and SwissTargetPrediction database to obtain gene targets (probability > 0), respectively. After correcting the results with the UniProt database, there were 680 gene targets corresponding to *Salvia miltiorrhiza* phenolic acid compounds and 151 gene targets corresponding to hawthorn triterpenic acid compounds. After removing the duplicate gene targets, a total of 747 unique gene targets were obtained. The abovementioned results were imported into Cytoscape 3.7.1 to construct the SHC–compound–target network diagram (Figure 2A).

#### 3.1.3 Collection of SHC-AS intersecting targets

The GeneCards and OMIM databases were queried using the term “atherosclerosis,” resulting in the identification of 5,304 disease-related targets after the application of a de-emphasis filter. The intersection of SHC-AS gene targets was performed using Venny 2.1, and 472 intersected targets were obtained (Figure 2B).

#### 3.1.4 Protein–protein interaction network construction and core target analysis

**FIGURE 3:**
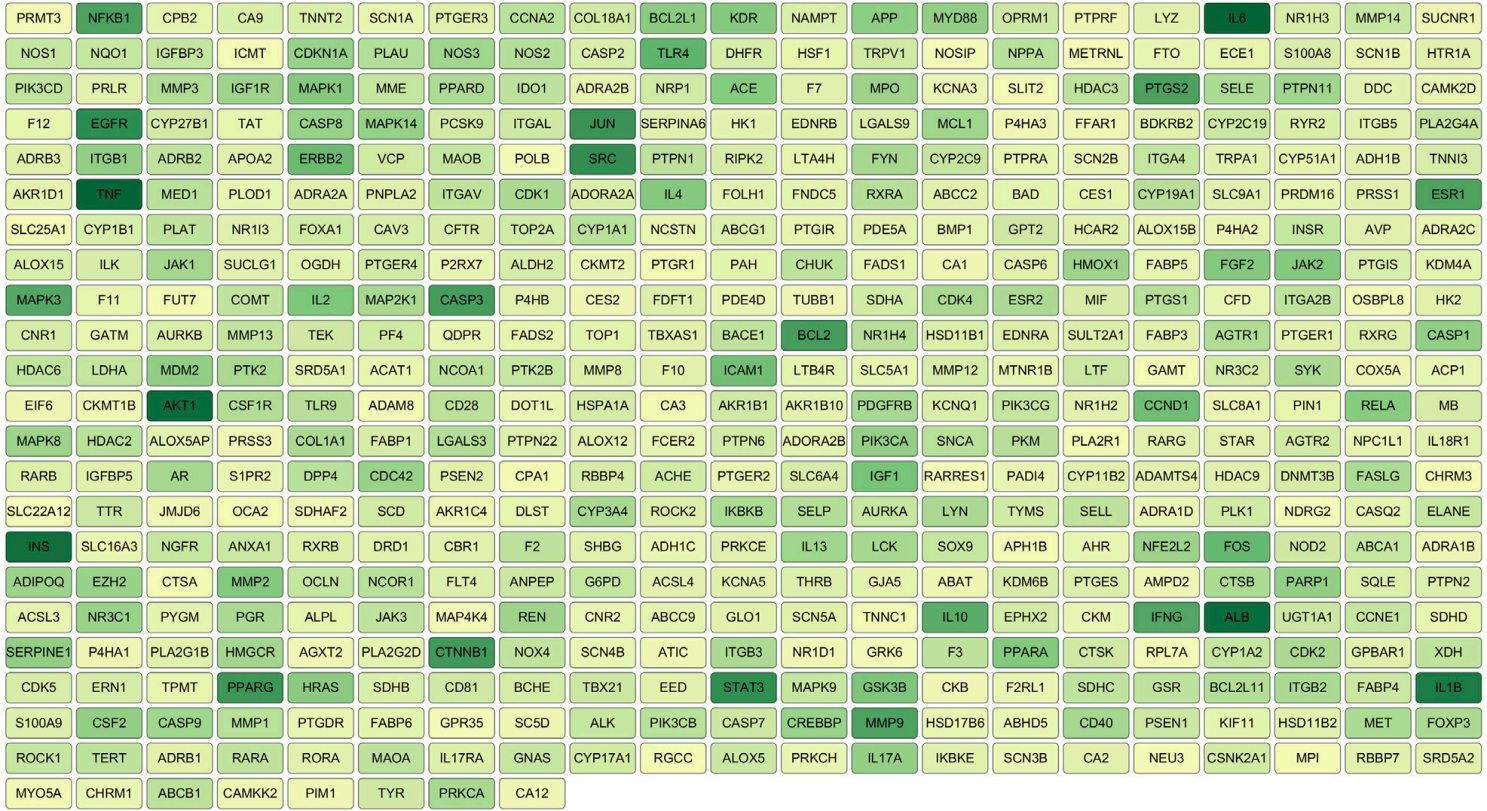
PPI network diagram.

**FIGURE 4:**
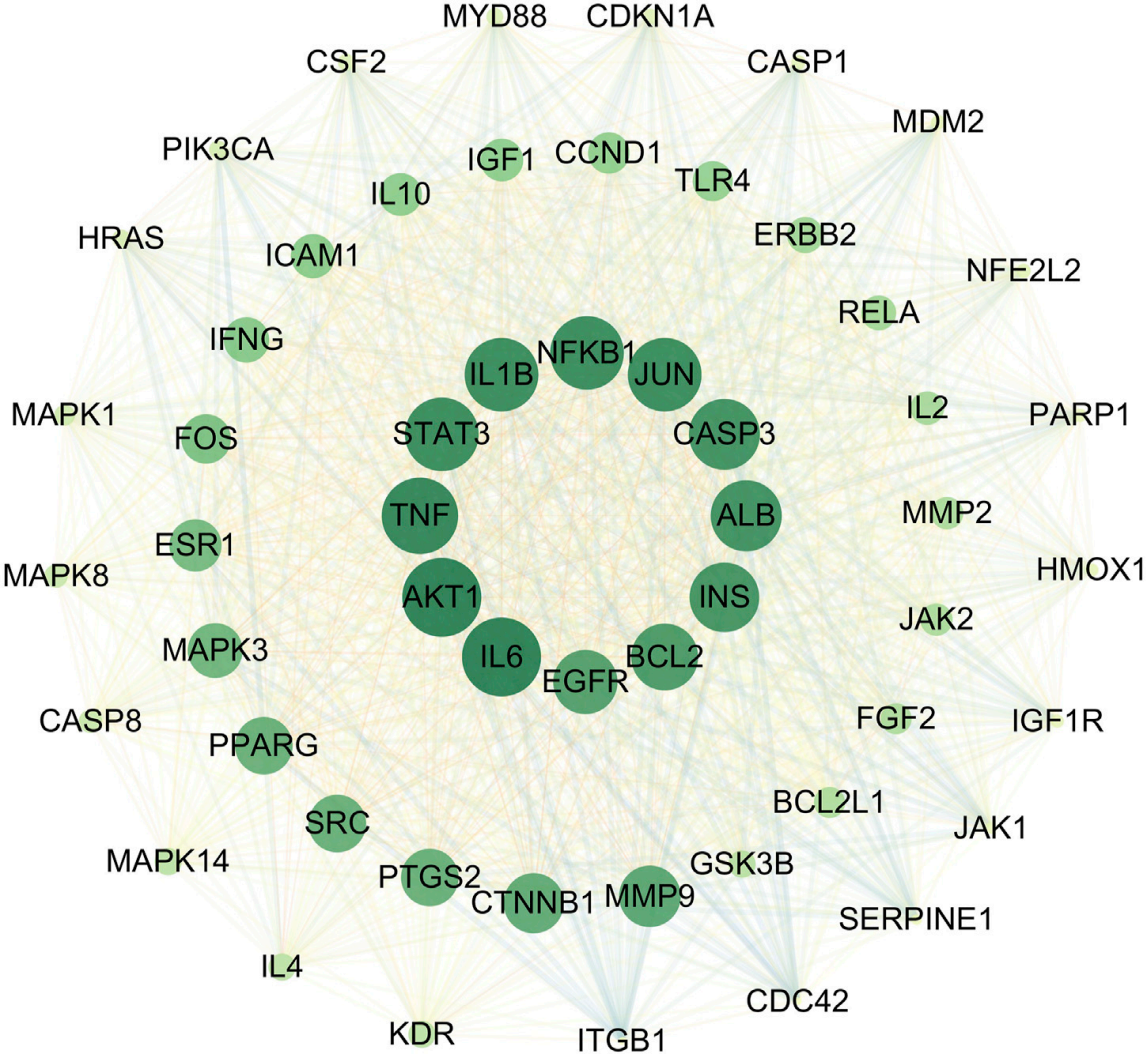
Key target diagram.

The 472 SHC-AS intersection targets were imported into the STRING database and subsequently imported into Cytoscape 3.7.1, following the download of the tsv file. The topological parameters in the network were then analyzed using the NetworkAnalyzer function in Cytoscape to construct the protein–protein interaction (PPI) network diagram (Figure 3). The application of the criterion degree ≥ 59 for screening yielded a total of 117 effective targets. Subsequently, utilizing a cutoff value of closeness ≥ 0.67 and betweenness ≥ 0.0018 for further screening, a total of 55 core targets were ultimately identified (Figure 4). The color intensity of each node represents the potential role it may play in SHC intervention in the context of atherosclerosis.

#### 3.1.5 Enrichment analysis

The 55 core targets were imported into the DAVID platform for GO analysis and KEGG analysis, and the final GO analysis yielded 580 BP-related entries, 57 CC-related entries, 73 MF-related entries, and 170 KEGG-related pathways.

**FIGURE 5:**
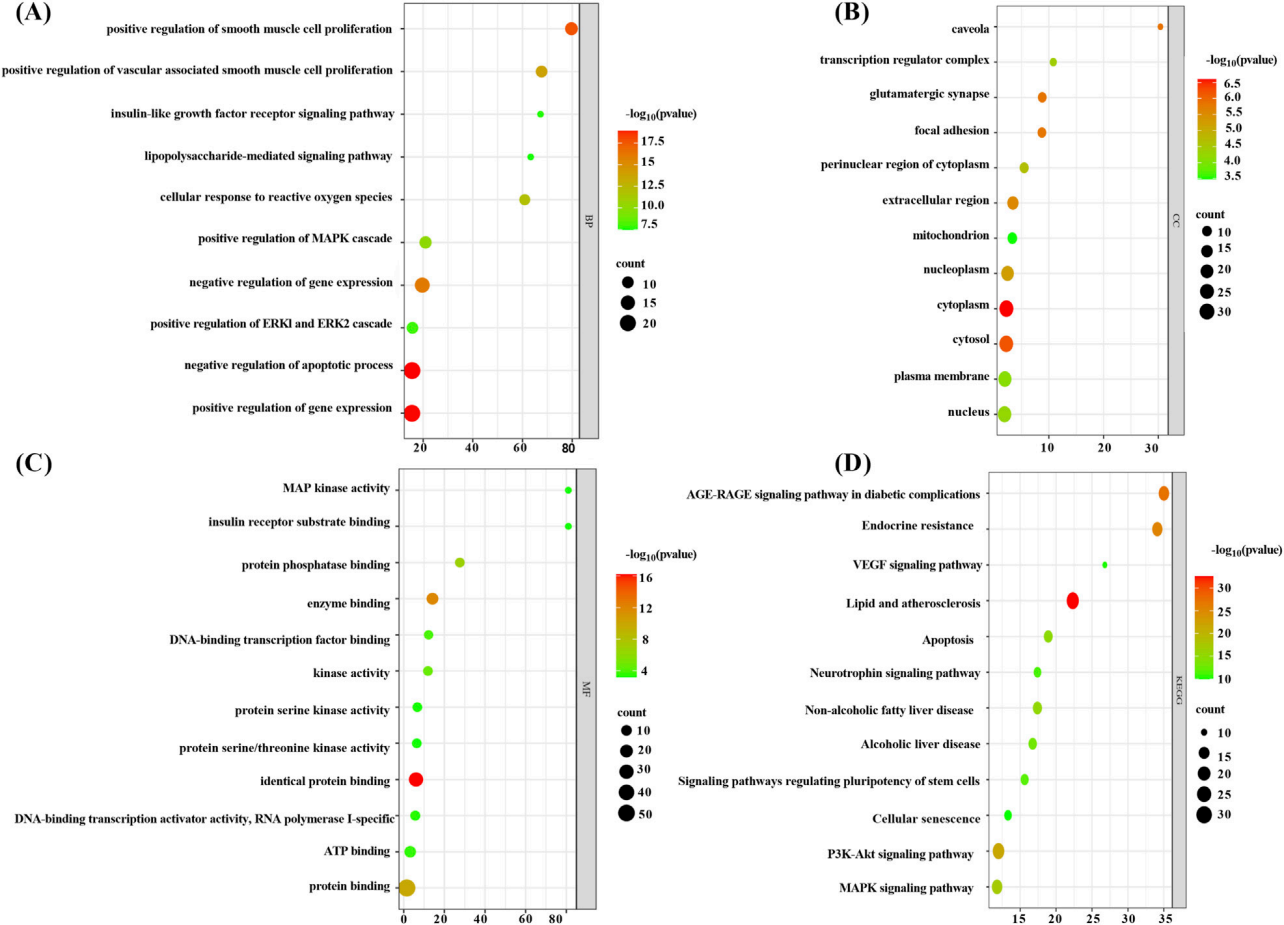
. Enrichment analysis bubble chart (note **(A)** BP enrichment analysis bubble chart, **(B)** CC enrichment analysis bubble chart, **(C)** MF enrichment analysis bubble chart, and **(D)** KEGG enrichment analysis bubble chart).

The BP category was primarily engaged in the negative regulation of the apoptotic process, the positive and negative regulation of gene expression, and the positive regulation of smooth muscle cell proliferation and vascular-associated smooth muscle cell proliferation, among other biological processes (Supplementary Table 2; Figure 5A). CC category was primarily associated with components such as the nucleus, cytoplasm, cytosol, and caveolae (Supplementary Table 3; Figure 5B). The MF category was predominantly comprised of protein binding, DNA-binding transcription factor binding, MAP kinase activity, and protein serine/threonine kinase activity (Supplementary Table 4; Figure 5C). The KEGG analysis was primarily associated with pathways such as lipid metabolism and atherosclerosis, the PI3K-Akt signaling pathway, the MAPK signaling pathway, and the VEGF signaling pathway, which plays a key role in angiogenesis and the regulation of vascular permeability (Supplementary Table 5; Figure 5D). The enrichment analysis revealed that core targets, including MAPK1, MAPK3, MAPK8, MAPK14, PIK3CA, AKT1, and NFKB1, frequently appeared in BP, CC, MF, and KEGG pathways. In light of the findings of previous research results (Reustle and Torzewski, 2018; Giulino-Roth et al., 2017; Monaco et al., 2004), the corresponding proteins of the aforementioned gene targets, including ERK1/2, JNK1, p-38 MAPK, PI3K, Akt, and NF-κB, were selected for validation.

### 3.2 Results of physiological characterization

**FIGURE 6:**
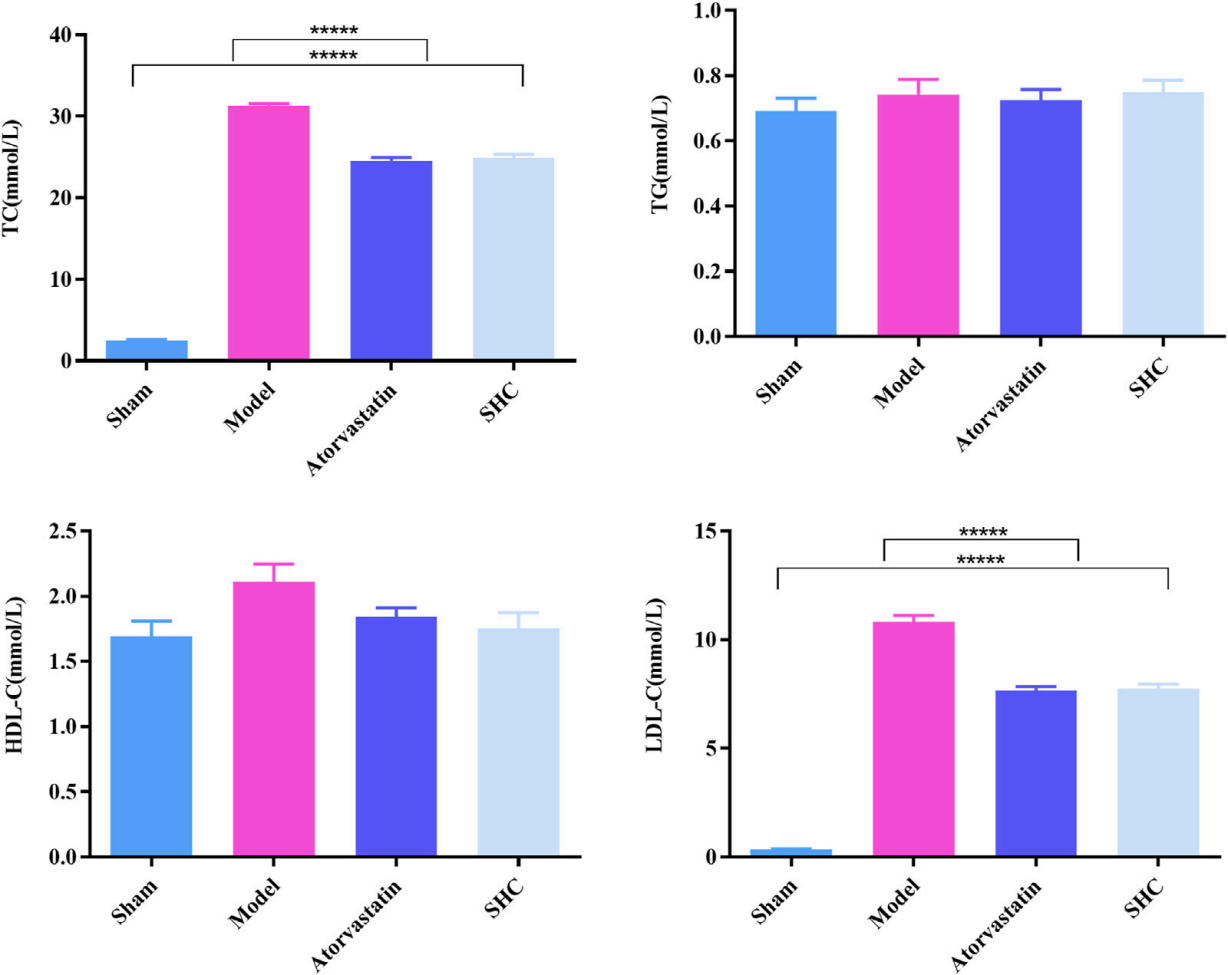
Levels of the four serum lipid profiles in mice from each group (*****P < 0.00001).

**FIGURE 7:**
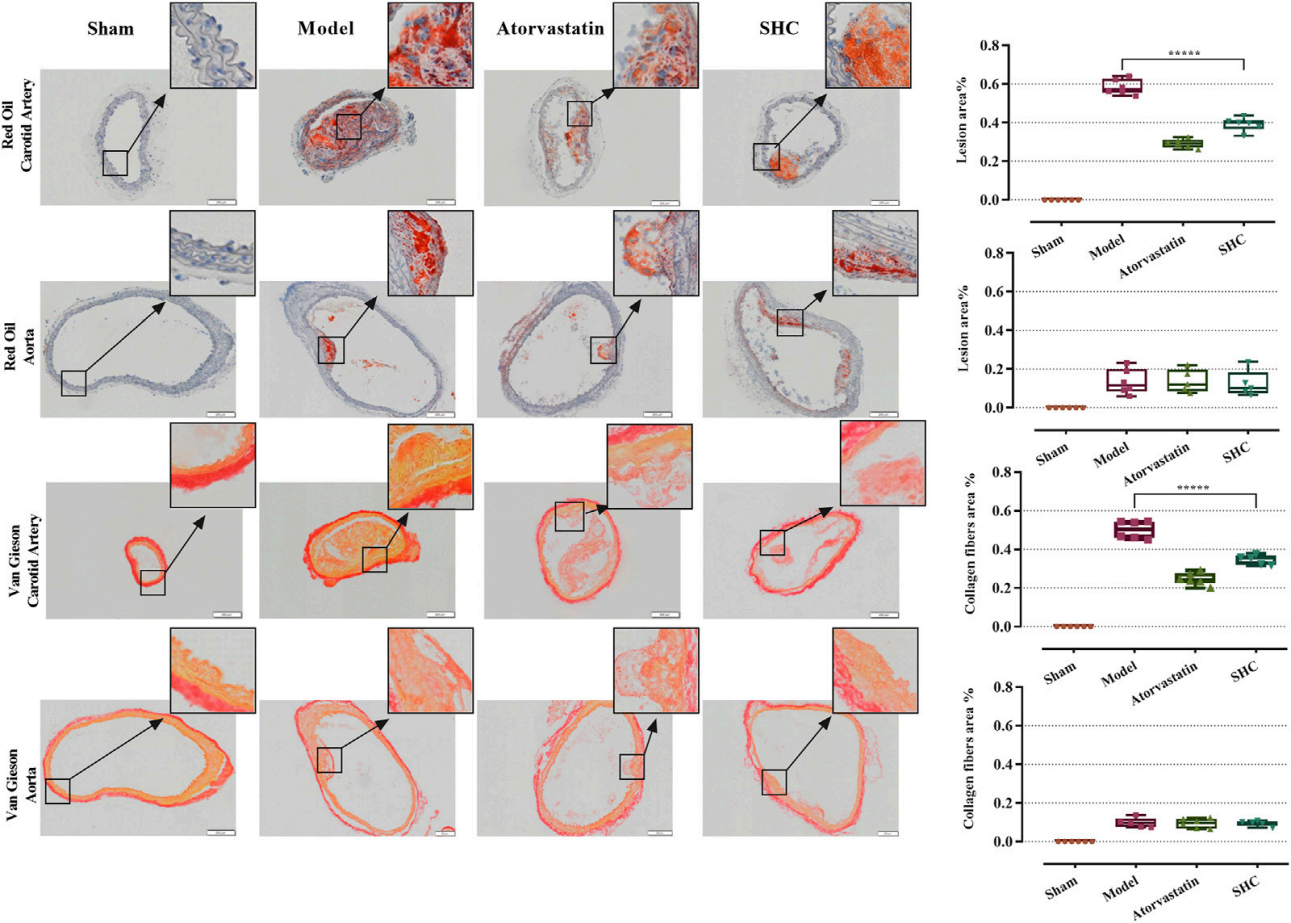
Oil red and VG staining plaque areas of the carotid artery and aorta in each group of mice (***P < 0.0001).

The results of the physiological characterization are divided into two categories, namely, serum lipid results and pathological results. Serum TC and LDL-C levels were markedly elevated in the model group (*P* < 0.00001), while atorvastatin and SHC demonstrated efficacy in markedly reducing TC and LDL-C levels (*P* < 0.00001), (Figure 6). Oil red staining, which causes lipid droplets to appear red and cell nuclei to appear blue, is a commonly used method for detecting vascular lipid deposition and plaque hyperplasia. The finding revealed the absence of lipid droplet deposition in the carotid arteries of the sham group, whereas the carotid arteries of the model group exhibited pronounced obstruction accompanied by a considerable accumulation of lipid droplets and foam cell hyperplasia. The deposition was significantly reduced in the atorvastatin and SHC groups (*P* < 0.00001). Oil red staining of the aorta demonstrated minimal lipid deposition in the model, atorvastatin, and SHC groups (Figure 7). VG staining enabled the differentiation of hyperplastic plaque formation through the visualization of collagen fibers in red and other tissue components in yellow. The findings revealed that the carotid arteries of the sham group exhibited no evidence of stenosis or plaque formation, whereas the arteries of the model group displayed more pronounced stenosis and plaque tissue formation. Additionally, the atorvastatin and SHC groups exhibited evidence of stenosis, although the degree of narrowing was less pronounced than that observed in the model group (*P* < 0.00001). A minimal amount of hyperplastic tissue was present in the aortas of the model, atorvastatin, and SHC groups, with no evidence of plaque formation (Figure 7).

### 3.3 Results of molecular biological characterization

**FIGURE 8:**
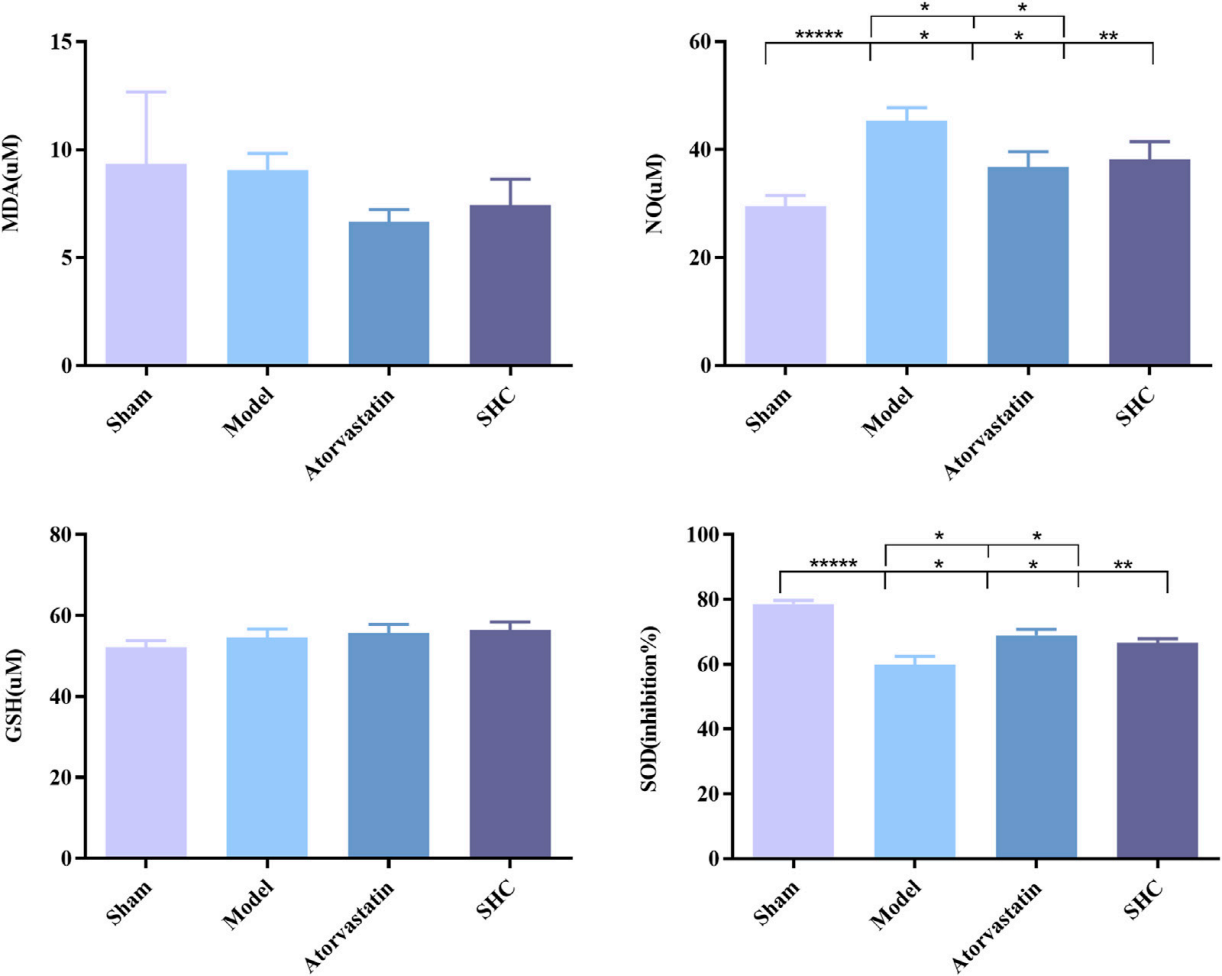
SOD, NO, MDA, and GSH levels in mice of each group (*P < 0.05, **P < 0.01, *****P < 0.00001).

**FIGURE 9:**
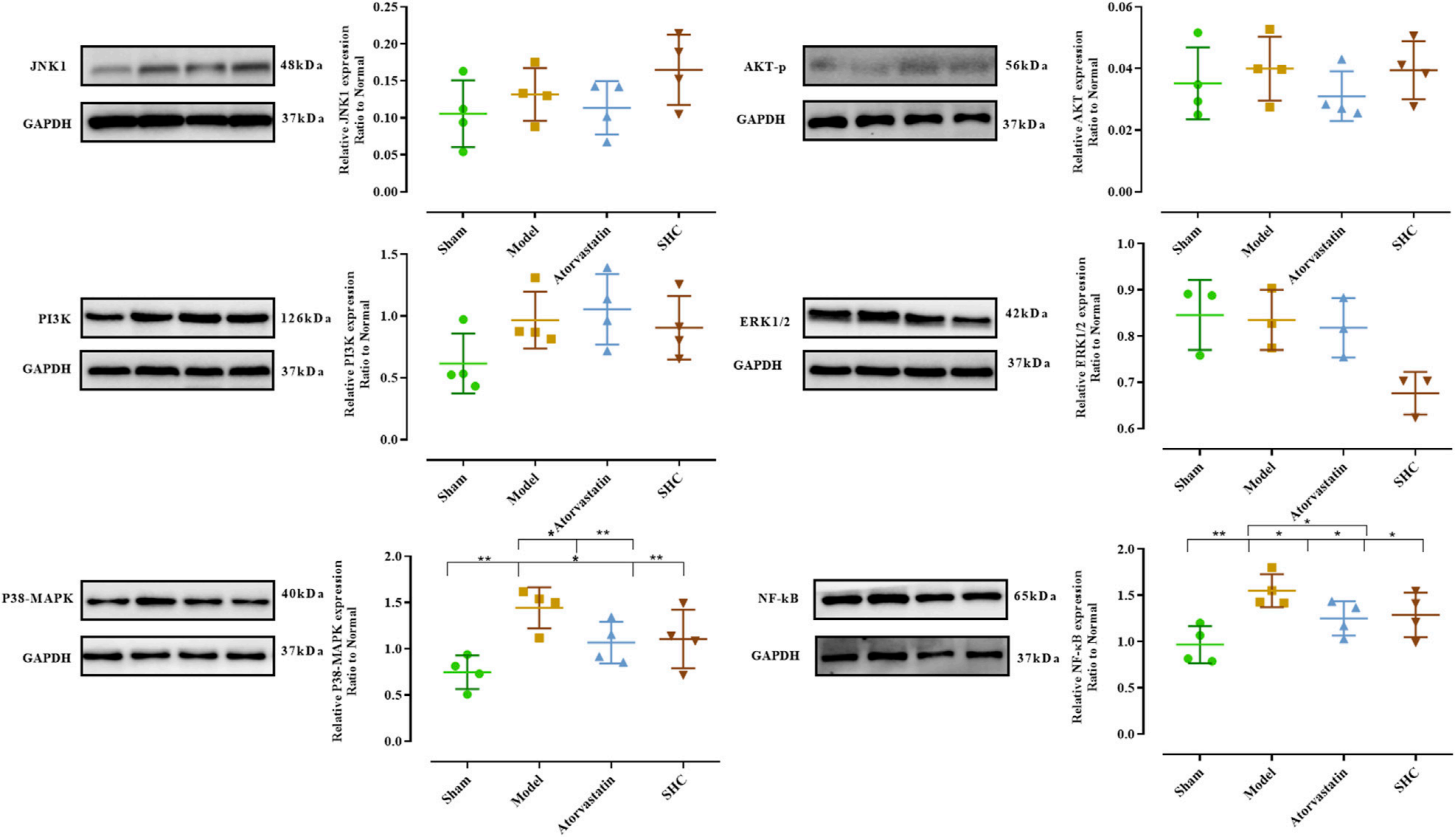
Expression of proteins levels in mice of each group (*P < 0.05, **P < 0.01).

Molecular biological characterization reveals differences in levels of oxidative stress biomarkers, including SOD, NO, MDA, and GSH, as well as Western blot results. SOD activity was markedly suppressed (*P* < 0.001) and NO levels were significantly elevated (*P* < 0.001) in the model group. Conversely, SOD activity was enhanced (*P* < 0.01, *P* < 0.05) and NO level was significantly diminished (*P* < 0.05, *P* < 0.05) in the atorvastatin and SHC groups. No significant difference was observed in the levels of MDA and GSH among the different groups (Figure 8). The expression of NF-κB and p38-MAPK was significantly higher in the model group (*P* < 0.01 and *P* < 0.01), whereas atorvastatin and SHC significantly reduced the expression of NF-κB (*P* < 0.05 and *P* < 0.05) and p38-MAPK (P < 0.05 and *P* < 0.01). In contrast, the expression of PI3K, Akt, ERK1/2, and JNK1 did not exhibit a significant difference between groups (Figure 9).

### 3.4 Molecular docking

**FIGURE 10:**
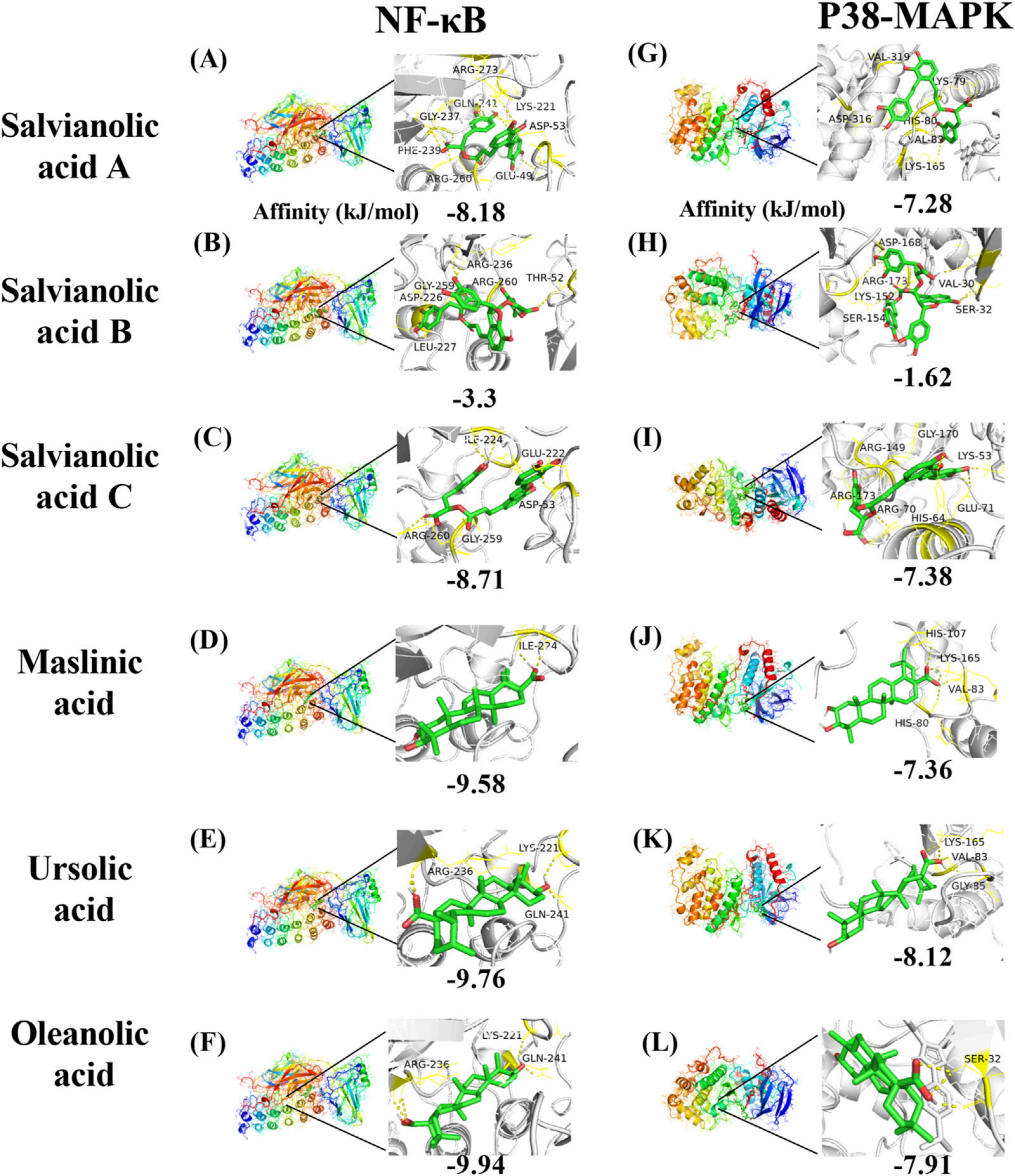
Molecular docking mode diagram. The intermolecular binding energies ≤ −5.0 kJ/mol as a threshold criterion was considered to be relatively strong interaction. [Note: **(A)** The binding of Salvianolic acid A to NF-κB, **(B)** the binding of Salvianolic acid B to NF-κB, **(C)** the binding of Salvianolic acid C to NF-κB, **(D)** the binding of Maslinic acid to NF-κB, **(E)** the binding of Ursolic acid to NF-κB, **(F)** the binding of Oleanolic acid to NF-κB, **(G)** the binding of Salvianolic acid A to P38-MAPK, **(H)** the binding of Salvianolic acid B to P38-MAPK, **(I)** the binding of Salvianolic acid C to P38-MAPK, **(J)** the binding of Maslinic acid to P38-MAPK, **(K)** the binding of Ursolic acid to P38-MAPK, **(L)** the binding of Oleanolic acid to P38-MAPK].

In conjunction with the network pharmacology and Western blot findings, the proteins corresponding to the core target NFKBIA (also known as NF-kappa-B inhibitor alpha or NF-κB) and MAPK14 (also known as mitogen-activated protein kinase or p38-MAPK) were identified as the research targets. The results were then visualized using PyMOL software (Figure 10). With regard to NF-κB, salvianolic acid C and oleanolic acid demonstrated superior binding affinity, with values of −8.71 kJ/mol and −9.94 kJ/mol, respectively. With regard to p38-MAPK, salvianolic acid C and ursolic acid demonstrated superior binding, with values of −7.38 kJ/mol and −8.12 kJ/mol, respectively. Nevertheless, salvianolic acid B demonstrated relatively weak binding affinity for NF-κB and p38-MAPK with a dissociation constant > −5 kJ/mol.

## 4 Discussion

### 4.1 AS and dyslipidemia

AS plaque formation was closely related to the accumulation of lipids, inflammatory cells, and smooth muscle cells within the arterial wall. LDL-C in blood could be deposited within the vessel wall and oxidized to ox-LDL. ox-LDL was highly cytotoxic and could activate endothelial cells, causing monocytes to cross the endothelial layer and transform into macrophages (Yu et al., 2013), forming foam cells. Foam cells released inflammatory factors that induced vascular smooth muscle cells (SMCs) to convert from a contractile to a synthetic phenotype, migrate to the endothelial layer, and proliferate. Collagen and elastin produced by SMC formed an extracellular matrix that provided structural support for plaque formation (Gui et al., 2022). Over time, plaques gradually increased in size, leading to the narrowing of the vessel lumen, which impeded the blood flow. This could ultimately lead to ischemia or vascular events in vital organs such as the heart and brain, such as myocardial infarction or stroke. Previous studies have shown that inflammatory responses are closely associated with the occurrence of AS (Bäck et al., 2019), NLRP3 inflammasome, toll-like receptor, interleukin-1β, preprotein convertase *bacillus subtilis* protease/kexin type 9 (Kong P. et al., 2022), NF-κB (Gan et al., 2023), and MAPK (Reustle and Torzewski, 2018; Wang Y et al., 2023). These inflammatory factors and their associated pathways were crucial for the development and regression of AS. In this study, the plaque area in ApoE-deficient mice was significantly increased; the levels of TC and LDL-C were both significantly elevated, and the expression of inflammation-related proteins such as NF-κB and p38-MAPK was significantly increased in the carotid artery.

In the inflammatory response, a large number of superoxide anion radicals (O^2-^) and reactive oxygen species (ROS) were produced due to the activation of immune cells. SOD, as an antioxidant enzyme, was able to catalyze the splitting of O^2-^ into oxygen O_2_ and hydrogen peroxide (H_2_O_2_) (Wu Q. et al., 2016). GSH, as a reducing agent, neutralized ROS through its oxidized form, glutathione disulfide (GSSG) neutralization of ROS (Perricone et al., 2009). Both SOD and GSH worked together to protect cells from oxidative damage and reduce oxidative stress during inflammation. NO had both anti-inflammatory and pro-inflammatory effects, and its overproduction may have led to tissue damage (Coleman J, 2001). MDA was the end product of lipid peroxidation, and it could be used as an important indicator for determining whether inflammatory responses were intense or not. In inflammatory responses, an increase in ROS led to an increase in lipid peroxidation and the subsequent elevation of MDA levels. The present study also showed that SOD levels were significantly decreased and NO levels were significantly increased in ApoE^−/−^ mice.

### 4.2 SHC and AS

Salvianolic acids are water-soluble extracts derived from the traditional Chinese medicinal herb Danshen (*Salvia miltiorrhiza*). To date, 197 polyphenolic acids have been identified in these extracts (Wu Y. et al., 2020). The majority of these acids are structured around the units of danshensu and caffeic acid, forming various dimers, trimers, and tetramers. Salvianolic acid A (Huang Q et al., 2022) and salvianolic acid C (Tang et al., 2016) have been demonstrated to possess potent anti-inflammatory and antioxidant activities, respectively. For example, salvianolic acids have been demonstrated to inhibit malignant hematopoiesis (Huang W et al., 2021), treat uterine fibroids (Tiwari et al., 2023), and combat breast cancer (Dalil et al., 2022). Given the established link between inflammation, oxidative stress, and AS, this study aims to investigate whether salvianolic acids can contribute to the treatment of AS through their anti-inflammatory and antioxidant properties.

In addition to its edible value, hawthorn fruit has medicinal properties. The lipophilic extract of hawthorn, which contains triterpenic acids, represents an important class of pentacyclic triterpenoid compounds. Major active constituents include maslinic acid, ursolic acid, and oleanolic acid (Zhang et al., 2022). Research indicates that hawthorn triterpenic acids may reduce triglyceride and cholesterol levels (Feng Y. et al., 2022) and have the potential to prevent non-alcoholic fatty liver disease (Liou et al., 2019). This may be related to their ability to improve myocardial energy metabolism and inhibit apoptosis (Zou et al., 2022). In addition, research has shown that maslinic acid can regulate postprandial hyperglycemia and prevent the development of diabetes (Mwakalukwa et al., 2020).

In this study, we used a variety of bioinformatics approaches, including network pharmacology and molecular docking methods, to enhance our research. Network pharmacology uses high-throughput technologies to rapidly screen and analyze large amounts of data, while molecular docking techniques predict the interactions between small and large molecules. These methods significantly improve the accuracy and reliability of our research, reduce experimental costs and time, and enable more comprehensive and efficient progress in drug discovery and disease research. In addition, an *in vivo* drug validation study was conducted to investigate the regulatory effects of the SHC on atherosclerosis.

### 4.3 Mechanism of action of SHC intervention in AS

To elucidate the mechanism of SHC in regulating dyslipidemia and anti-inflammation, this study identified that SHC’s mechanism of action in intervening with AS primarily involves the lipid and AS signaling pathway, the PI3K-Akt signaling pathway, and the MAPK signaling pathway, among others, through network pharmacology. The Kyoto Encyclopedia of Genes and Genomes (https://www.kegg.jp/) indicates that the PI3K-Akt pathway and the MAPK inflammatory factor play a significant role in the “lipid and AS pathway.” Prior research has demonstrated that the inhibition of PI3K-Akt (Giulino-Roth et al., 2017) and mitogen-activated protein kinase (MAPK) activity in carotid arteries and vascular endothelial cells can impede the progression of plaques (Wang Y et al., 2023). Moreover, the generation of pro-inflammatory cytokines in AS plaques was found to be closely associated with NF-κB (Monaco et al., 2004). Both the ROS and PI3K/Akt signaling pathways activated NF-κB, which was produced by the cells. In the absence of stimulation, NF-κB is bound to inhibitory proteins, such as IKKβ and IKKα, and is located in the cytoplasm. The presence of moderate amounts of ROS in the cytoplasm, along with pro-inflammatory cytokines (TNF-α and IL-1β) and lipopolysaccharide (LPS), stimulates the cell. This stimulation (Gloire et al., 2006) results in the phosphorylation of the inhibitory protein IKKβ, leading to the release of NF-κB and its subsequent translocation into the nucleus, where it regulates the expression of multiple target genes. Consequently, Akt may also phosphorylate IKKα in response to PI3K, resulting in its rapid degradation and the release of NF-κB (Poma, 2020). This, in turn, continues to promote the production of multiple inflammatory mediators, including cytokines, chemokines, and coagulation factors. These further exacerbate inflammatory responses, induce the expression of adhesion molecules, promote leukocyte binding and transport, and promote plaque formation (Mussbacher et al., 2019). MAPK is a serine/threonine protein kinase that includes extracellular signal-regulated kinase (ERK), c-Jun amino-terminal kinase (JNK), and p38-MAPK. Upon the stimulation of the cell by a drug or cytokine, the MAPKs are activated, resulting in the generation of an inflammatory response throughout the regulation of downstream signals (Qi R. et al., 2017). The results of network pharmacological studies and research indicated a strong association between MAPKs and the development of AS inflammatory responses (Huang et al., 2021). Additionally, evidence has demonstrated that p38-MAPK/NF-κB activation increases inflammatory responses in AS (Lima et al., 2020).

To elucidate the potential targets of SHC intervention in AS, in this study, we combined the results of network pharmacological prediction with those of previous literature studies. The selected target proteins for validation were MAPKs (JNK1, ERK1/2, p38-MAPK), PI3K/Akt (PI3K, Akt), and NFKBIA (NF-κB). Their expression was then detected using Western blotting in mice, with the expression in arteries serving as the expression site. The results demonstrated a significant increase in NF-κB expression in the model group, which was significantly decreased in the atorvastatin and SHC groups. Similarly, p38-MAPK expression was significantly elevated in the model group, whereas atorvastatin and SHC were able to significantly downregulate p38-MAPK expression. No significant differences were observed in the expression of PI3K, Akt, ERK, and JNK between the groups. This suggests that NF-κB and p38-MAPK are closely associated with AS plaque formation and that SHC may reduce the inflammatory response in AS and halt the progression of the plaque.

### 4.4 Discussion of molecular docking

To further elucidate the pharmacological basis of SHC intervention in AS, this study employed molecular docking techniques to investigate the binding conformation between targets and compounds. The results of network pharmacology and Western blot validation indicated that NF-κB and p38-MAPK should be considered core targets for further investigation. A literature search conducted on PubMed and a network pharmacology analysis identified the following compounds as potential core compounds: salvianolic acid A (Dawuti et al., 2023), B (Zhao L et al., 2023), and C (Song et al., 2018); maslinic acid (Lee et al., 2020); ursolic acid (Chen et al., 2018); and oleanolic acid (Feng et al., 2011). The intermolecular binding energies of ≤ −5.0 kJ/mol represent a relatively strong interaction, which may contribute to maintaining a stable binding state within organisms (Wang R et al., 2003). The results of the molecular docking analysis demonstrated that, in addition to salvianolic acid B, the remaining five components of SHC exhibited lower binding energies with NF-κB and p38-MAPK, indicating a higher probability of interaction.

#### 4.4.1 NF-κB molecular docking

Duan et al. demonstrated that salvianolic acid C can inhibit the nuclear translocation of the NF-κB p65 subunit induced by LPS. This action inhibits the transcription and expression of downstream genes, reduces the levels of inflammatory cytokines and oxidative stress in cells, and consequently suppresses the occurrence of inflammatory responses (Duan et al., 2019). Furthermore, Song et al. demonstrated through *in vivo* and *in vitro* experiments that salvianolic acid C can significantly activate the nuclear factor erythroid 2-related factor (Nrf2) signaling pathway. As a pivotal antioxidant and anti-inflammatory transcription factor, Nrf2, when activated, induces the expression of a range of antioxidant enzymes and anti-inflammatory proteins, thereby indirectly inhibiting NF-κB activity and reducing the production of inflammatory mediators (Song et al., 2018). These studies confirm that salvianolic acid C can bind to NF-κB and inhibit NF-κB-mediated inflammatory responses through both direct and indirect mechanisms.

Fontana et al. employed molecular docking techniques to simulate the binding of oleanolic acid and its derivatives with NF-κB (p65 subunit), thereby predicting their potential interaction patterns. The experimental results indicated that some oleanolic acid derivatives exhibit a high binding affinity with NF-κB (p65 subunit) and can significantly inhibit the DNA binding ability of HL60 and HL60R cells and NF-κB (p65 subunit), thereby suppressing the activation of the NF-κB signaling pathway, which, in turn, inhibits tumor cell proliferation or induces apoptosis (Fontana et al., 2022). Iskender et al. also corroborated this finding, demonstrating that oleanolic acid administration resulted in a notable reduction in blood glucose and lipid levels in diabetic rats. It was observed that oleanolic acid could directly decrease the levels of NF-κB and MDA in diabetic rats and improve inflammation and oxidative damage in pancreatic tissue (Iskender et al., 2022). Moreover, the findings of this study indicate that salvianolic acid A, salvianolic acid C, maslinic acid, ursolic acid, and oleanolic acid can interact with NF-κB, which may represent one of the mechanisms by which they exert their protective effects against atherosclerosis.

#### 4.4.2 p38-MAPK molecular docking

In their study, Liu et al. employed cancer cell lines A549, BXPC-3, PANC-1, and U2OS to elucidate the molecular mechanisms underlying the anti-tumor effects of oleanolic acid. The experiments demonstrated that oleanolic acid activates p38 MAPK in a dose- and time-dependent manner, promotes the mitochondrial translocation of Bax and Bim, and inhibits the function of Bcl-2 by enhancing their phosphorylation. *In vivo* experiments demonstrated that A549 tumors with p38-MAPK knockdown exhibited resistance to the inhibitory effects of oleanolic acid (Liu J. et al., 2014). Wang et al. observed that mice with spinal cord injury exhibited elevated expression of MAPKs, and treatment with oleanolic acid resulted in a notable reduction in the phosphorylation levels of p38-MAPK. In lipopolysaccharide (LPS)-stimulated mouse neurons, oleanolic acid has been demonstrated to inhibit apoptosis and inflammatory responses by blocking p38 MAPK (Wang J. L et al., 2020). The precise sites of action of oleanolic acid on p38-MAPK remain undetermined. However, the aforementioned studies have confirmed that oleanolic acid can interact with p38-MAPK.

Lin et al. demonstrated that isosalvianolic acid C is involved in pseudo-allergic reactions through the activation of the p38-MAPK signaling pathway (Lin et al., 2019). This finding suggests that salvianolic acid C may target p38-MAPK. Further investigation will be conducted in subsequent studies. The present study has identified salvianolic acid A, salvianolic acid C, maslinic acid, ursolic acid, and oleanolic acid as potential interactants with p38-MAPK, with the possibility of regulating downstream signaling molecules.

## 5 Conclusion

It is hypothesized that SHC may reduce lipid deposition and plaque formation in AS by regulating blood lipids, a process that may be closely linked to the inhibition of inflammatory regulator expression, including NF-κβ and p38-MAPK.

## Data Availability

The original contributions presented in the study are included in the article/Supplementary Material; further inquiries can be directed to the corresponding author.
