# Supplementary material for: Mechanism of salvianolic phenolic acids and hawthorn triterpenic acids combination in intervening atherosclerosis: network pharmacology, molecular docking, and experimental validation
Source: Front Pharmacol. 2025 Jan 30;16:1501846. doi: 10.3389/fphar.2025.1501846 (PMC11821658; doi:10.3389/fphar.2025.1501846)
Supplement: Supplementary file 1 [file Table1.docx]

**Supplementary Table 1.** Core ingredients of SHC(Note OB:oral bioavailability, DL:drug-likeness)

|  | Compound | Molecular formula | CAS | OB (%) | DL |
| --- | --- | --- | --- | --- | --- |
| Phenolic acids in Salvia miltiorrhiza | Salvianolic acid A | C26H22O10 | 96574-01-5 | 2.96 | 0.7 |
|  | Salvianolic acid B | C36H30O16 | 121521-90-2 | 3.01 | 0.41 |
|  | Salvianolic acid C | C26H20O10 | 115841-09-3 | 2.5 | 0.81 |
|  | Salvianolic acid D | C20H18O10 | 142998-47-8 | 1.57 | 0.5 |
|  | Salvianolic acid G | C18H12O7 | 136112-79-3 | 45.56 | 0.61 |
|  | Tanshinol A | C18H12O4 | N/A | 21.31 | 0.41 |
|  | Tanshinol B | C18H16O4 | 96839-29-1 | 38.35 | 0.03 |
|  | Protocatechuic Aldehyde | C7H6O3 | 99-50-3 | 38.35 | 0.03 |
|  | Vanillic acid | C8H8O4 | 121-34-6 | 31.11 | 0.05 |
|  | Caffeic acid | C9H8O4 | 331-39-5 | 30.68 | 0.06 |
|  | Ferulic acid | C10H10O4 | 1135-24-6 | 39.56 | 0.06 |
|  | Rosmarinic acid | C18H16O8 | 20283-92-5 | 1.38 | 0.35 |
|  | Danshensu | C9H10O5 | 76822-21-4 | 36.91 | 0.06 |
|  | Propanoic acid | C3H6O2 | 79-09-4 | 1.57 | 0.5 |
|  | Acrylic acid | C3H4O2 | 9003-01-4 | 1.58 | 0.7 |
|  | Lithospermic acid | C27H22O12 | 28831-65-4 | 2.67 | 0.76 |
|  | 4-Methyl-1,2-benzenedio | C7H8O2 | 452-86-8 | 45.22 | 0.02 |
|  | 4-Hydroxybenzoic acid | C7H6O3 | 99-96-7 | 30.15 | 0.03 |
|  | 4-Coumaric acid | C9H8O3 | 501-98-4 | 43.29 | 0.04 |
| triterpenic acids in Hawthorn Fruit | Euscaphic acid | C30H48O5 | 53155-25-2 | 11.4 | 0.71 |
|  | Maslinic acid | C30H48O4 | 4373-41-5 | 15.54 | 0.74 |
|  | Oleanic acid | C30H48O3 | 508-02-1 | 29.02 | 0.76 |
|  | Ursolic acid | C30H48O3 | 77-52-1 | 16.77 | 0.75 |
