# Supplementary material for: Mechanism of salvianolic phenolic acids and hawthorn triterpenic acids combination in intervening atherosclerosis: network pharmacology, molecular docking, and experimental validation
Source: Front Pharmacol. 2025 Jan 30;16:1501846. doi: 10.3389/fphar.2025.1501846 (PMC11821658; doi:10.3389/fphar.2025.1501846)
Supplement: Supplementary file 2 [file DataSheet3.docx]

**Supplementary 3**

**Reagents**

| **Name** | **Article No.** | **Manufacturer** | **City** | **Country** |
| --- | --- | --- | --- | --- |
| Water | W6-4 | Thermo Fisher Scientific | Waltham | USA |
| Methanol | A456-4 | Thermo Fisher Scientific | Waltham | USA |
| Acetonitrile | A955-4 | Thermo Fisher Scientific | Waltham | USA |
| Formic acid | A117-50 | Thermo Fisher Scientific | Waltham | USA |
| Atorvastatin Calcium Tablets | H20170219 | Pfizer Inc. | Vega Baja | USA |
| JNK1 | ab110724 | Abcam plc | Cambridge | UK |
| PI3K | ab32089 | Abcam plc | Cambridge | UK |
| ERK1/2 | ab184699 | Abcam plc | Cambridge | UK |
| Akt phospho-t308 | ab38449 | Abcam plc | Cambridge | UK |
| NF-kβ | 8242S | Cell Signaling Technology | Danvers | USA |
| One Step Western Kit HRP | CW2030M | CoWin Biosciences Inc. | TaiZhou | China |
| eECL Western Blot Kit | CW0049M | CoWin Biosciences Inc. | TaiZhou | China |
| Total Protein Extraction Kit | BC3710-100T | Beijing Solarbio Science & Technology Co.,Ltd. | Beijing | China |
| Total Glutathione Assay Kit | S0052 | Beyotime Biotech Inc. | Shanghai | China |
| Total Superoxide Dismutase Assay Kit | S0101S | Beyotime Biotech Inc. | Shanghai | China |
| Lipid Peroxidation MDA Assay Kit | S0131S | Beyotime Biotech Inc. | Shanghai | China |
| Total Nitric Oxide Assay Kit | S0023 | Beyotime Biotech Inc. | Shanghai | China |
| Salvia miltiorrhiza | ACS610 | Guiyang Xintian Pharmaceutical Co. | Guiyang | China |
| Hawthorn | ACH017 | Guiyang Xintian Pharmaceutical Co. | Guiyang | China |
