# Supplementary material for: Mechanism of salvianolic phenolic acids and hawthorn triterpenic acids combination in intervening atherosclerosis: network pharmacology, molecular docking, and experimental validation
Source: Front Pharmacol. 2025 Jan 30;16:1501846. doi: 10.3389/fphar.2025.1501846 (PMC11821658; doi:10.3389/fphar.2025.1501846)
Supplement: Supplementary file 3 [file Table2.docx]

**Supplementary Table 2**.Enrichment Analysis-BP

| Term | | Fold Enrichment | PValue | Count | Class | Genes |
| --- | --- | --- | --- | --- | --- | --- |
| GO:0043066 | negative regulation of apoptotic process | 15.56923077 | 6.11E-20 | 22 | BP | IL10, GSK3B, CDKN1A, SRC, IGF1, TNF, MMP9, EGFR, IL2, RELA, NFKB1, IGF1R, IL4, IL6, MAPK8, ERBB2, MDM2, KDR, BCL2, AKT1, CTNNB1, BCL2L1 |
| GO:0010628 | positive regulation of gene expression | 15.50805501 | 6.63E-20 | 22 | BP | GSK3B, CSF2, STAT3, IGF1, MAPK14, FGF2, TNF, RELA, INS, IL4, IL6, MAPK8, IFNG, IL1B, ERBB2, MDM2, AKT1, CTNNB1, PPARG, TLR4, MYD88, NFE2L2 |
| GO:0048661 | positive regulation of smooth muscle cell proliferation | 79.73333333 | 1.89E-18 | 12 | BP | IL6, PIK3CA, HMOX1, AKT1, IGF1, PTGS2, TNF, FGF2, TLR4, EGFR, MYD88, IGF1R |
| GO:0010629 | negative regulation of gene expression | 19.67612903 | 1.11E-16 | 17 | BP | GSK3B, CDKN1A, SRC, STAT3, IGF1, FGF2, ESR1, TNF, NFKB1, INS, IFNG, PIK3CA, KDR, AKT1, CTNNB1, PPARG, HRAS |
| GO:1904707 | positive regulation of vascular associated smooth muscle cell proliferation | 67.69811321 | 1.73E-14 | 10 | BP | IL10, JUN, SRC, MMP2, MDM2, IGF1, JAK2, TNF, FGF2, MMP9 |
| GO:0034614 | cellular response to reactive oxygen species | 60.92830189 | 1.47E-12 | 9 | BP | JUN, MAPK8, MMP2, MAPK1, AKT1, FOS, MMP9, EGFR, MAPK3 |
| GO:0043410 | positive regulation of MAPK cascade | 20.99361702 | 8.06E-11 | 11 | BP | IL6, ERBB2, KDR, CTNNB1, IGF1, JAK2, HRAS, TNF, FGF2, IGF1R, INS |
| GO:0070374 | positive regulation of ERK1 and ERK2 cascade | 15.73684211 | 1.06E-08 | 10 | BP | SRC, KDR, IGF1, HRAS, TNF, FGF2, TLR4, EGFR, ICAM1, MAPK3 |
| GO:0048009 | insulin-like growth factor receptor Signaling pathway | 67.275 | 2.42E-08 | 6 | BP | PIK3CA, MAPK1, AKT1, IGF1, IGF1R, MAPK3 |
| GO:0031663 | lipopolysaccharide-mediated Signaling pathway | 63.31764706 | 3.32E-08 | 6 | BP | MAPK1, AKT1, MAPK14, TLR4, MYD88, MAPK3 |
