# Supplementary material for: Mechanism of salvianolic phenolic acids and hawthorn triterpenic acids combination in intervening atherosclerosis: network pharmacology, molecular docking, and experimental validation
Source: Front Pharmacol. 2025 Jan 30;16:1501846. doi: 10.3389/fphar.2025.1501846 (PMC11821658; doi:10.3389/fphar.2025.1501846)
Supplement: Supplementary file 4 [file Table5.docx]

**Supplementary** Table 5.Enrichment Analysis-KEGG

| Term | | Fold Enrichment | PValue | Count | CLASS | Genes |
| --- | --- | --- | --- | --- | --- | --- |
| hsa05417 | Lipid and atherosclerosis | 22.32323232 | 2.57E-33 | 30 | KEGG | GSK3B, SRC, TNF, RELA, ICAM1, CDC42, MAPK8, CASP8, CASP3, CASP1, AKT1, MAPK1, JAK2, HRAS, MAPK3, JUN, STAT3, FOS, MAPK14, MMP9, NFKB1, IL6, PIK3CA, IL1B, BCL2, PPARG, TLR4, MYD88, BCL2L1, NFE2L2 |
| hsa04933 | AGE-RAGE Signaling pathway in diabetic complications | 35.00990099 | 6.92E-28 | 22 | KEGG | JUN, MMP2, STAT3, SERPINE1, MAPK14, TNF, RELA, NFKB1, ICAM1, CDC42, IL6, MAPK8, PIK3CA, CCND1, IL1B, CASP3, BCL2, AKT1, MAPK1, JAK2, HRAS, MAPK3 |
| hsa01522 | Endocrine resistance | 34.09366391 | 2.97E-26 | 21 | KEGG | CDKN1A, JUN, SRC, MMP2, FOS, IGF1, MAPK14, ESR1, MMP9, EGFR, IGF1R, MAPK8, PIK3CA, CCND1, ERBB2, MDM2, BCL2, AKT1, MAPK1, HRAS, MAPK3 |
| hsa04151 | PI3K-Akt Signaling pathway | 11.98794576 | 2.16E-22 | 27 | KEGG | ITGB1, GSK3B, CDKN1A, FGF2, RELA, EGFR, IGF1R, INS, CCND1, ERBB2, KDR, AKT1, MAPK1, JAK2, HRAS, JAK1, MAPK3, IGF1, IL2, NFKB1, IL4, IL6, PIK3CA, BCL2, MDM2, TLR4, BCL2L1 |
| hsa04010 | MAPK Signaling pathway | 11.78666667 | 1.25E-17 | 22 | KEGG | JUN, FOS, IGF1, MAPK14, FGF2, TNF, EGFR, RELA, NFKB1, INS, IGF1R, CDC42, MAPK8, IL1B, CASP3, ERBB2, KDR, AKT1, MAPK1, HRAS, MYD88, MAPK3 |
| hsa04932 | Non-alcoholic fatty liver disease | 17.40359004 | 5.15E-16 | 17 | KEGG | GSK3B, JUN, FOS, MAPK14, TNF, RELA, NFKB1, INS, CDC42, IL6, MAPK8, CASP8, PIK3CA, IL1B, CASP3, AKT1, PPARG |
| hsa04210 | Apoptosis | 18.90909091 | 1.47E-15 | 16 | KEGG | JUN, PARP1, FOS, TNF, RELA, NFKB1, MAPK8, CASP8, PIK3CA, CASP3, BCL2, AKT1, MAPK1, HRAS, BCL2L1, MAPK3 |
| hsa04936 | Alcoholic liver disease | 16.74242424 | 9.09E-14 | 15 | KEGG | GSK3B, MAPK14, TNF, RELA, NFKB1, IL6, MAPK8, CASP8, CCND1, IL1B, CASP3, AKT1, CTNNB1, TLR4, MYD88 |
| hsa04550 | Signaling pathways regulating pluripotency of stem cells | 15.62626263 | 2.06E-12 | 14 | KEGG | GSK3B, STAT3, IGF1, MAPK14, FGF2, IGF1R, PIK3CA, AKT1, MAPK1, CTNNB1, JAK2, HRAS, JAK1, MAPK3 |
| hsa04722 | Neurotrophin Signaling pathway | 17.41212121 | 4.75E-12 | 13 | KEGG | GSK3B, JUN, MAPK14, RELA, NFKB1, CDC42, MAPK8, PIK3CA, BCL2, AKT1, MAPK1, HRAS, MAPK3 |
| hsa04370 | VEGF Signaling pathway | 26.78787879 | 6.87E-11 | 10 | KEGG | CDC42, PIK3CA, SRC, KDR, MAPK1, AKT1, MAPK14, PTGS2, HRAS, MAPK3 |
| hsa04218 | Cellular senescence | 13.30862768 | 1.16E-10 | 13 | KEGG | CDKN1A, SERPINE1, MAPK14, RELA, NFKB1, IL6, PIK3CA, CCND1, MDM2, AKT1, MAPK1, HRAS, MAPK3 |
