# Supplementary material for: Mechanism of salvianolic phenolic acids and hawthorn triterpenic acids combination in intervening atherosclerosis: network pharmacology, molecular docking, and experimental validation
Source: Front Pharmacol. 2025 Jan 30;16:1501846. doi: 10.3389/fphar.2025.1501846 (PMC11821658; doi:10.3389/fphar.2025.1501846)
Supplement: Supplementary file 5 [file Table3.docx]

**Supplementary Table 3**.Enrichment Analysis-CC

| Term | | Fold Enrichment | PValue | Count | Class | Genes |
| --- | --- | --- | --- | --- | --- | --- |
| GO:0005737 | cytoplasm | 2.243997851 | 2.49502E-07 | 34 | CC | ITGB1, GSK3B, SRC, PTGS2, FGF2, RELA, EGFR, CDC42, MAPK8, CASP8, CCND1, CASP3, ERBB2, CASP1, AKT1, MAPK1, JAK2, HRAS, JAK1, MAPK3, STAT3, MAPK14, ESR1, NFKB1, PIK3CA, ALB, BCL2, MDM2, CTNNB1, PPARG, TLR4, MYD88, BCL2L1, NFE2L2 |
| GO:0005829 | cytosol | 2.218481147 | 6.4727E-07 | 33 | CC | GSK3B, CDKN1A, SRC, RELA, CDC42, MAPK8, CASP8, CCND1, CASP3, ERBB2, CASP1, AKT1, MAPK1, HMOX1, JAK2, HRAS, JAK1, MAPK3, PARP1, STAT3, FOS, MAPK14, ESR1, NFKB1, PIK3CA, IL1B, BCL2, MDM2, CTNNB1, PPARG, MYD88, BCL2L1, NFE2L2 |
| GO:0005901 | caveola | 30.38109091 | 1.43745E-06 | 6 | CC | SRC, MAPK1, JAK2, PTGS2, IGF1R, MAPK3 |
| GO:0098978 | glutamatergic synapse | 8.790824916 | 1.48627E-06 | 10 | CC | CDC42, GSK3B, SRC, CTNNB1, AKT1, JAK2, MAPK14, HRAS, RELA, MAPK3 |
| GO:0005925 | focal adhesion | 8.730198537 | 1.57342E-06 | 10 | CC | CDC42, ITGB1, SRC, MAPK1, CTNNB1, JAK2, EGFR, JAK1, ICAM1, MAPK3 |
| GO:0005576 | extracellular region | 3.434321319 | 2.8758E-06 | 19 | CC | IL10, CSF2, MMP2, SERPINE1, IGF1, MAPK14, FGF2, TNF, MMP9, IL2, NFKB1, INS, IL4, IL6, IFNG, IL1B, ALB, KDR, MAPK1 |
| GO:0005654 | nucleoplasm | 2.450087977 | 6.01602E-06 | 26 | CC | GSK3B, CDKN1A, SRC, RELA, MAPK8, CASP8, CCND1, CASP3, ERBB2, AKT1, MAPK1, HMOX1, JAK2, HRAS, MAPK3, JUN, PARP1, STAT3, FOS, MAPK14, ESR1, NFKB1, MDM2, CTNNB1, PPARG, NFE2L2 |
| GO:0048471 | perinuclear region of cytoplasm | 5.48935611 | 2.17482E-05 | 11 | CC | ITGB1, CDKN1A, PIK3CA, SRC, ERBB2, HMOX1, CTNNB1, PPARG, HRAS, TLR4, EGFR |
| GO:0005667 | transcription regulator complex | 10.80628234 | 4.04121E-05 | 7 | CC | JUN, PARP1, STAT3, CTNNB1, ESR1, RELA, NFKB1 |
| GO:0005634 | nucleus | 1.906505705 | 5.90846E-05 | 31 | CC | GSK3B, CDKN1A, FGF2, RELA, EGFR, MAPK8, CCND1, CASP3, ERBB2, KDR, AKT1, MAPK1, HMOX1, JAK2, JAK1, MAPK3, JUN, PARP1, MMP2, STAT3, FOS, MAPK14, ESR1, NFKB1, ALB, BCL2, MDM2, CTNNB1, PPARG, MYD88, NFE2L2 |
| GO:0005886 | plasma membrane | 1.969446612 | 7.70769E-05 | 29 | CC | ITGB1, GSK3B, CSF2, SRC, SERPINE1, TNF, EGFR, IGF1R, ICAM1, CDC42, MAPK8, ERBB2, CASP1, KDR, AKT1, MAPK1, JAK2, HRAS, JAK1, MAPK3, MMP2, STAT3, ESR1, PIK3CA, MDM2, CTNNB1, TLR4, MYD88, NFE2L2 |
| GO:0005739 | mitochondrion | 3.32453015 | 0.00033268 | 13 | CC | GSK3B, PARP1, SRC, MMP2, MAPK14, NFKB1, MAPK8, CASP8, BCL2, AKT1, MAPK1, BCL2L1, MAPK3 |
