# Supplementary material for: Mechanism of salvianolic phenolic acids and hawthorn triterpenic acids combination in intervening atherosclerosis: network pharmacology, molecular docking, and experimental validation
Source: Front Pharmacol. 2025 Jan 30;16:1501846. doi: 10.3389/fphar.2025.1501846 (PMC11821658; doi:10.3389/fphar.2025.1501846)
Supplement: Supplementary file 6 [file DataSheet2.docx]

**Supplementary 2**

**Chemical Components Analysis of SHC**

1. ***Experiment***

**1.1 Sample preparation**

50 mg of SHC sample and 2 mL 80% methanol were charged into a 15 mL centrifuge tube, and ultrasonicated for 30 minutes. After that, 1 mL suspension was transferred into a 1.5 mL tube and centrifuged at 4°C and 12000 rpm for 10 min. Finally, 100 μL of supernatant was pipetted into an injection vial for detection (the details of the regents can be found in Supplementary 3.Regents).

**1.2 Chromatographic conditions**

A Vanquish Flex UHPLC chromatograph (Thermo Fisher Scientific, Inc., Waltham, MA, USA) equipped with an ACQUITY UPLC HSS T3 column (2.1 mm (inner diameter) ×100 mm (length), 1.7 μm (particle dimension)) (Waters Corp., MA, USA) was used for separation. The mobile phase was consisted of water (0.1% formic acid, phase A) and acetonitrile (phase B) with a flow rate of 0.3 mL/min and the column temperature was 40°C. The elution gradient was shown in Table 1 and the injection volume was 6.0 µL.

Table 1 Elution gradient

| Time (min) | Mobile phase | |
| --- | --- | --- |
|  | A (v%) | B (v%) |
| 0 | 98 | 2 |
| 1.0 | 98 | 2 |
| 14.0 | 70 | 30 |
| 25.0 | 0 | 100 |
| 28.0 | 0 | 100 |
| 28.1 | 98 | 2 |
| 30.0 | 98 | 2 |

**1.3 MS conditions**

The MS data was collected by a hybrid quadrupole orbitrap mass spectrometer (Q Exactive, Thermo Fisher Scientific, Inc., Waltham, MA, USA) equipped with a HESI-II spray probe. The parameters were set as follows: positive ion source voltage 3.7 kV and negative ion source voltage 3.5 kV, heated capillary temperature 320°C, sheath gas pressure 30 psi, auxiliary gas pressure 10 psi, desolvation temperature 300°C. Both the sheath gas and the auxiliary gas were nitrogen. The collision gas was also nitrogen with a pressure of 1.5 mTorr. The data was acquired in “Full scan/dd-MS^2^” mode. The parameters of the full scan were set as follows: resolution 7000, auto gain control target 1×10^6^, maximum isolation time 50 ms. The dd-MS^2^ data was collected with the parameters of resolution 17500, auto gain control target 1×10^5^, maximum isolation time 50 ms, loop count of top 10 peaks, isolation window of m/z 2, collision energy 10 V, 30 V, 60 V and intensity threshold 1×10^5^.

**1.4 Data analysis**

The MS data was processed by Progenesis QI 3.0 (Waters Corp., MA, USA) with the steps of raw data introduction, peak extraction and adduct deconvolution. The identification was finally determined by in consideration of retention time error of reference substance, mass error of mother ion, match degree of daughter ions, isotope distribution and peak area after searching the reference substance database (TCM Pro 2.0, Beijing Hexin Technology Co., Ltd) and theoretical database constructed by literature and public databases.

1. ***Results***

**2.1 Chromatogram**

**2.1.1 Chromatogram of Salvianolic Phenolic Acids**

**
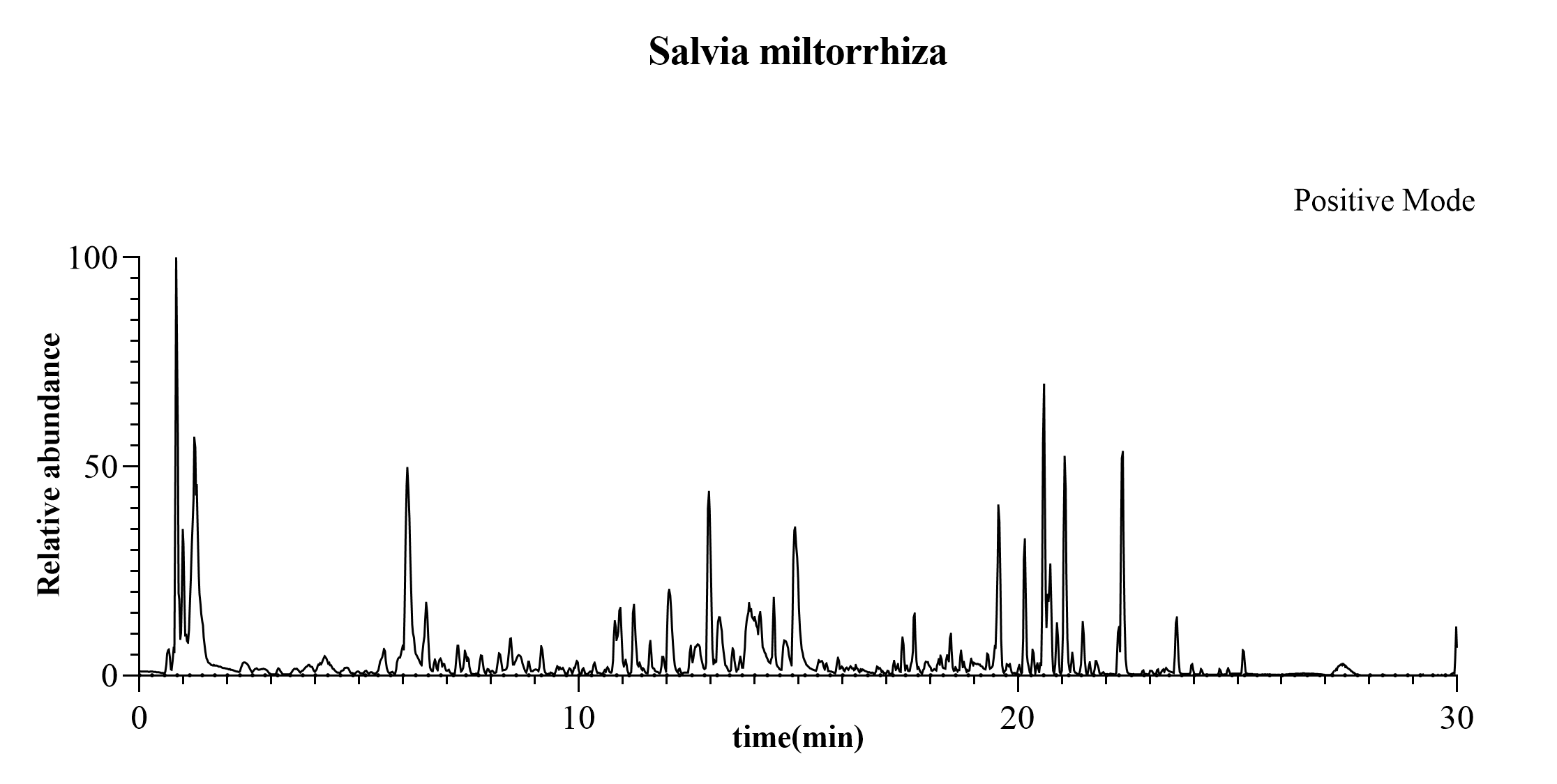
**

FIGURE 1.Base peak ion (BPI) chromatogram of Salvianolic Phenolic Acids detected in positive mode.

**
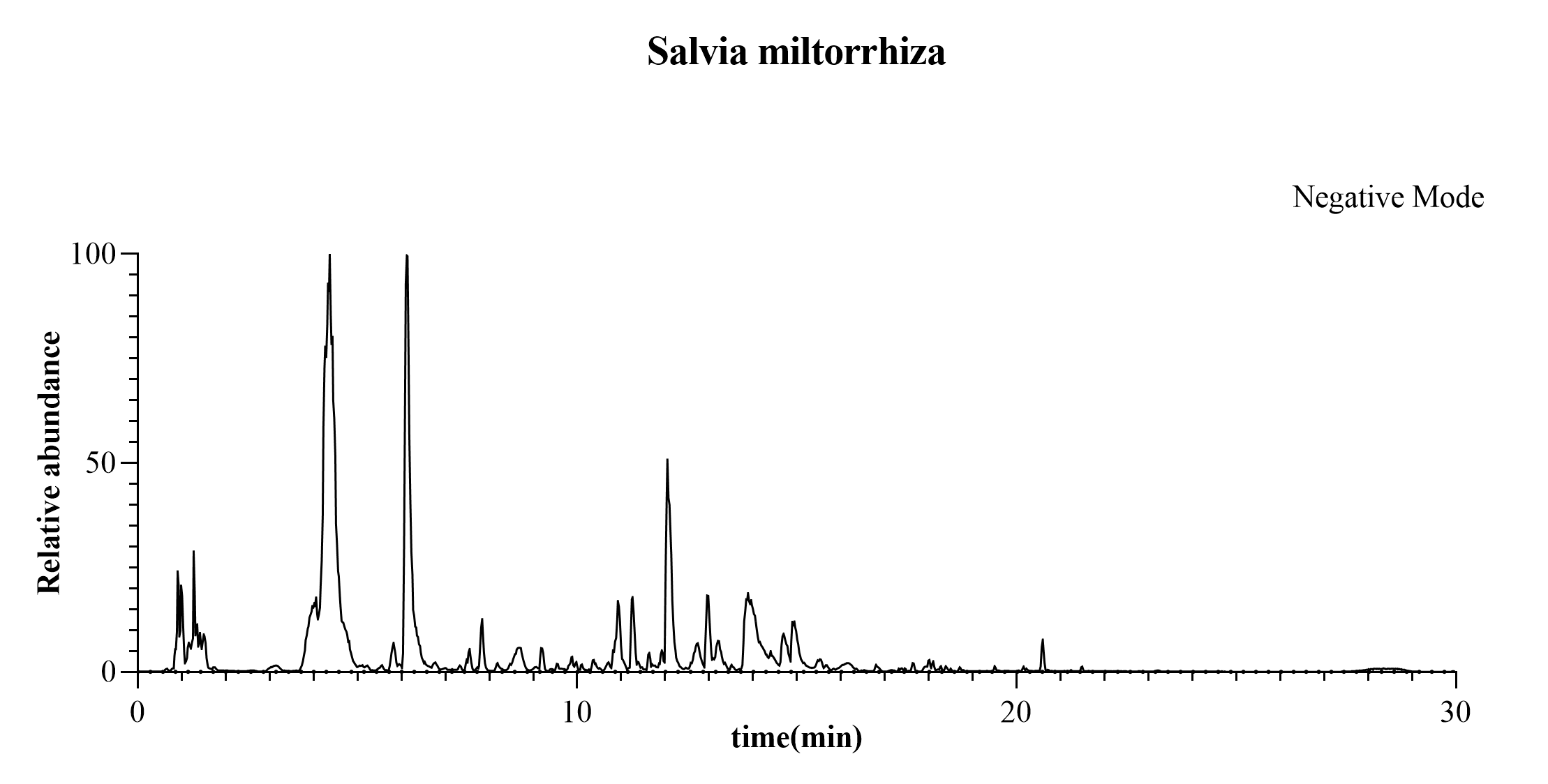
**

FIGURE 2.Base peak ion (BPI) chromatogram of Salvianolic Phenolic Acids detected in negative mode.

**2.1.2 Chromatogram of Hawthorn Triterpenic Acids**

**
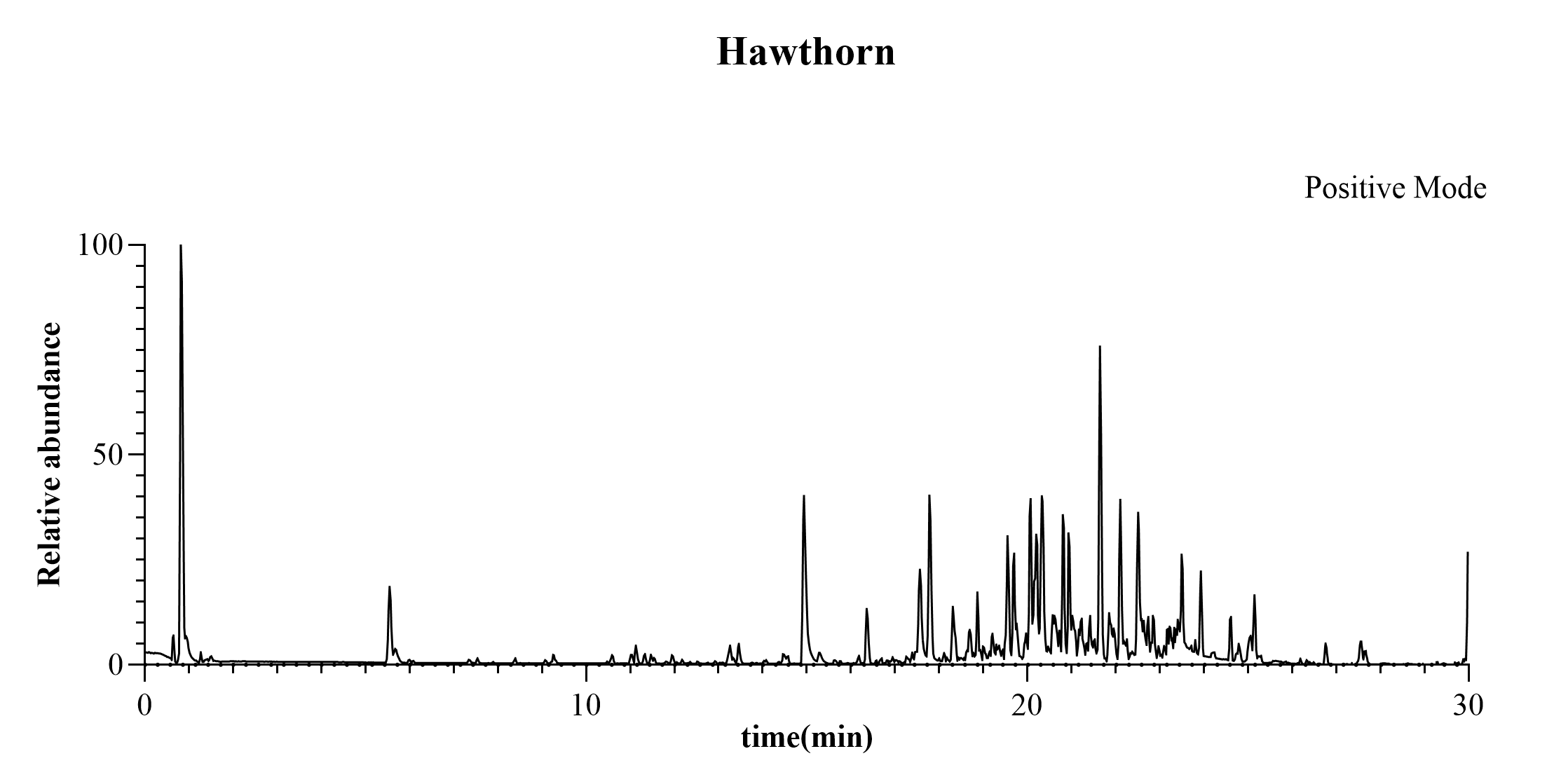
**

FIGURE 3.Base peak ion (BPI) chromatogram of Hawthorn Triterpenic Acids detected in positive mode.


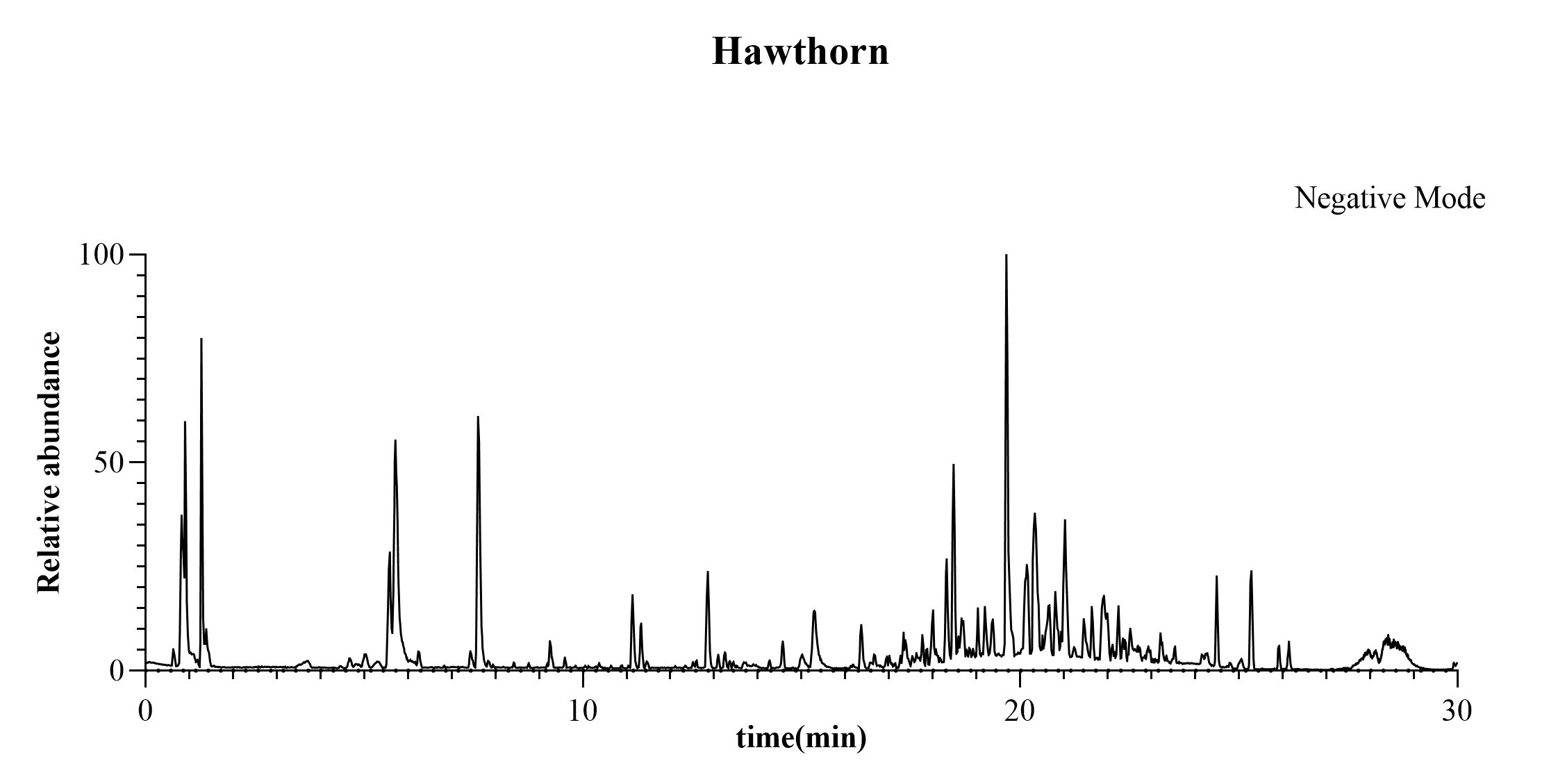


FIGURE 4.Base peak ion (BPI) chromatogram of Hawthorn Triterpenic Acids detected in negative mode.

**Reference**

Yang Yu, Changliang Yao, De-an Guo. Insight into chemical basis of traditional Chinese medicine based on the state-of-the-art techniques of liquid chromatography-mass spectrometry. Acta Pharmaceutica Sinica B, 2021, 11(6), 1469 – 1492.
