# Supplementary material for: Mechanism of salvianolic phenolic acids and hawthorn triterpenic acids combination in intervening atherosclerosis: network pharmacology, molecular docking, and experimental validation
Source: Front Pharmacol. 2025 Jan 30;16:1501846. doi: 10.3389/fphar.2025.1501846 (PMC11821658; doi:10.3389/fphar.2025.1501846)
Supplement: Supplementary file 7 [file Table4.docx]

**Supplementary Table 4.** Enrichment Analysis-MF

| Term | | Fold Enrichment | PValue | Count | Class | Genes |
| --- | --- | --- | --- | --- | --- | --- |
| GO:0042802 | identical protein binding | 6.061862145 | 6.25E-17 | 30 | MF | FGF2, TNF, RELA, EGFR, IGF1R, INS, CDC42, CASP8, ERBB2, CASP1, KDR, AKT1, MAPK1, HMOX1, JAK2, MAPK3, JUN, PARP1, STAT3, FOS, MMP9, ESR1, NFKB1, ALB, BCL2, MDM2, PPARG, TLR4, MYD88, BCL2L1 |
| GO:0019899 | enzyme binding | 14.15249267 | 1.23E-12 | 15 | MF | JUN, PARP1, SRC, PTGS2, MAPK14, ESR1, EGFR, RELA, MAPK8, CCND1, MDM2, AKT1, HMOX1, CTNNB1, PPARG |
| GO:0005515 | protein binding | 1.513564372 | 1.83E-10 | 55 | MF | ITGB1, GSK3B, CDKN1A, CSF2, SERPINE1, FGF2, TNF, IGF1R, ICAM1, CASP8, CCND1, CASP3, CASP1, KDR, AKT1, JAK2, HRAS, JAK1, IL10, PARP1, MMP2, FOS, MMP9, IFNG, PIK3CA, IL1B, PPARG, TLR4, SRC, PTGS2, EGFR, RELA, INS, CDC42, MAPK8, ERBB2, MAPK1, HMOX1, MAPK3, JUN, STAT3, IGF1, MAPK14, ESR1, NFKB1, IL2, IL4, IL6, ALB, BCL2, MDM2, CTNNB1, MYD88, NFE2L2, BCL2L1 |
| GO:0019903 | protein phosphatase binding | 27.60531154 | 1.75E-07 | 7 | MF | MAPK8, STAT3, CTNNB1, PPARG, MAPK14, EGFR, JAK1 |
| GO:0016301 | kinase activity | 11.92656664 | 2.31E-05 | 7 | MF | GSK3B, CDKN1A, PIK3CA, ERBB2, AKT1, JAK2, EGFR |
| GO:0140297 | DNA-binding transcription factor binding | 12.2435518 | 1.18E-04 | 6 | MF | STAT3, BCL2, CTNNB1, PPARG, RELA, MAPK3 |
| GO:0005524 | ATP binding | 3.188673235 | 2.53E-04 | 14 | MF | GSK3B, SRC, MAPK14, EGFR, IGF1R, MAPK8, PIK3CA, ERBB2, KDR, AKT1, MAPK1, JAK2, JAK1, MAPK3 |
| GO:0001228 | DNA-binding transcription activator activity, RNA polymerase II-specific | 5.695445325 | 4.26E-04 | 8 | MF | JUN, STAT3, PPARG, FOS, ESR1, RELA, NFKB1, NFE2L2 |
| GO:0106310 | protein serine kinase activity | 6.712767014 | 5.35E-04 | 7 | MF | GSK3B, MAPK8, PIK3CA, MAPK1, AKT1, MAPK14, MAPK3 |
| GO:0004707 | MAP kinase activity | 80.9958042 | 5.87E-04 | 3 | MF | MAPK1, MAPK14, MAPK3 |
| GO:0043560 | insulin receptor substrate binding | 80.9958042 | 5.87E-04 | 3 | MF | PIK3CA, JAK2, IGF1R |
| GO:0004674 | protein serine/threonine kinase activity | 6.431603998 | 6.69E-04 | 7 | MF | GSK3B, MAPK8, PIK3CA, MAPK1, AKT1, MAPK14, MAPK3 |
