# Supplementary material for: Mechanism of salvianolic phenolic acids and hawthorn triterpenic acids combination in intervening atherosclerosis: network pharmacology, molecular docking, and experimental validation
Source: Front Pharmacol. 2025 Jan 30;16:1501846. doi: 10.3389/fphar.2025.1501846 (PMC11821658; doi:10.3389/fphar.2025.1501846)
Supplement: Supplementary file 8 [file DataSheet1.docx]

**Supplementary 1**

**Extraction of SHC components**

1. ***Extraction of total phenolic acids from Salvia miltorrhiza***

This product is an extract derived from the dried roots and rhizomes of Salvia miltiorrhiza Bge., a plant in the Lamiaceae family, which has undergone a series of processing steps, including extraction and separation techniques, prior to its final form.

- 1. **Preparation method**

Salvia miltiorrhiza slices were extracted by decoction with water on two occasions. In the initial decoction, ten times the volume of water was employed, while in the subsequent decoction, six times the volume of water was utilized, with each decoction lasting 1.5 hours. Subsequently, the mixture was filtered, and the resulting filtrates were combined. The combined filtrate was concentrated to a density whereby 1 mL is equivalent to 1.0 g of the original herbal slices (with a relative density of 1.20 at 50°C). Subsequently, a 4% gelatin solution (comprising 2.0% gelatin relative to the herbal slices) was incorporated into the mixture, which was then permitted to stand for 12 hours prior to filtration. The filtrate was concentrated to a relative density of 1.16 at 50°C, and ethanol was added to achieve a 70% ethanol content. Subsequently, the mixture was filtered once more after a 12-hour period of standing. The precipitate was then washed with 60% ethanol, with the same volume of ethanol used in the initial precipitation step, and allowed to stand for 12 hours before filtration. The filtrates were then combined and concentrated to a relative density of 1.23 at 50°C. Subsequently, the pH was adjusted to a range of 2-3 with dilute hydrochloric acid. Thereafter, the extract was shaken with an equal volume of water-saturated ethyl acetate in three extractions. The ethyl acetate fractions were then combined, and the solvent was recovered. The resulting paste was adjusted to pH 5 with 1% sodium hydroxide, and then spray-dried to obtain the final product, with an average yield of total phenolic acids greater than 2.0%.

**1.2 Description**

The product is a brownish-yellow amorphous powder with a fragrant aroma and a sour taste. The substance is readily soluble in water, methanol, ethanol, and ethyl acetate.

1. ***Extraction of total total triterpenoic acid from Hawthorn***

This product is an extract derived from the dried fruits of Crataegus pinnatifida Bge., a plant in the Rosaceae family, which has undergone a series of extraction, separation, and processing procedures.

**2.1 Preparation method**

The hawthorn fruit should be pulverized into a coarse powder and extracted with 80% ethanol by percolation, twice. In the initial extraction, the powder should be added in a quantity eight times greater than the volume of the solvent. In the subsequent extraction, the ratio should be four times greater. Each extraction should be conducted for a period of 0.5 hours. The mixture should be filtered, the filtrates combined, and the ethanol recovered under reduced pressure. This will yield a clear paste with a relative density of 1.11 at 50°C. The clear paste should be stirred with water three times, with the volume of water added each time being ten times that of the paste. This process should continue until the pH of the resulting wash solution reaches 6. The mixture should then be left to stand, filtered, and the resulting aqueous liquid discarded. The residue should then be spray-dried to produce a dry powder, with a yield of greater than 3.0%. Subsequently, acetic ether is added to the dry powder in a ratio of four times the powder's volume. The mixture is then warm-soaked at 60°C for 0.5 hours, filtered, and the acetic ether is recovered to obtain a black paste, which is subsequently discarded. Subsequently, the residue is refluxed with acetic ether three times, with the addition of ten times the volume of acetic ether each time. The initial reflux lasts for one hour, while the second and third refluxes each last for half an hour. The filtrates should be filtered, combined, the acetic ether recovered, and the mixture dried under vacuum. This process yields the final product, which has a total triterpenic acid yield of greater than 5.0%.

**2.2 Description**

The product is a yellowish-brown amorphous powder that is odorless and tasteless. The substance is soluble in methanol, ethanol, ethyl acetate, and diethyl ether, but insoluble in water.

1. ***SHC mixture preparation***

**3.1 The chemical composition of SHC**

Extraction of total phenolic acids from Salvia miltorrhiza: 97.5g

Extraction of total total triterpenoic acid from Hawthorn: 52.5g

**3.2 Preparation method**

The two aforementioned components, Salvia miltiorrhiza total phenolic acid extract and Crataegus pinnatifida total triterpenic acid extract, were combined with 100 grams of microcrystalline cellulose in a ratio of 2:1:2, forming a solution. Subsequently, 40ml of ethanol was added to the mixture and thoroughly stirred to form granules, which were then dried for storage.

**3.3**  **Description**

The color of SHC ranges from light brownish-yellow to brownish-yellow. The product has a distinctive aromatic quality and a slightly acidic flavor.
